# Room Temperature Phosphorescent Nanofiber Membranes by Bio‐Fermentation

**DOI:** 10.1002/advs.202405327

**Published:** 2024-07-01

**Authors:** Xiaolin Nie, Junyi Gong, Zeyang Ding, Bo Wu, Wen‐Jin Wang, Feng Gao, Guoqing Zhang, Parvej Alam, Yu Xiong, Zheng Zhao, Zijie Qiu, Ben Zhong Tang

**Affiliations:** ^1^ School of Science and Engineering Shenzhen Institute of Aggregate Science and Technology Clinical Translational Research Center of Aggregation‐Induced Emission School of Medicine, The Second Affiliated Hospital The Chinese University of Hong Kong, Shenzhen (CUHK‐Shenzhen) Guangdong 518172 P. R. China; ^2^ Hefei National Research Center for Physical Sciences at the Microscale University of Science and Technology of China Hefei 230026 China; ^3^ Center for AIE Research Shenzhen Key Laboratory of Polymer Science and Technology Guangdong Research Center for Interfacial Engineering of Functional Materials College of Materials Science and Engineering Shenzhen University Shenzhen 518061 P. R. China; ^4^ Department of Chemistry Hong Kong Branch of Chinese National Engineering Research Center for Tissue Restoration and Reconstruction The Hong Kong University of Science and Technology Kowloon Hong Kong China

**Keywords:** anti‐counterfeiting, bacterial cellulose nanofibers, information encryption, organic room temperature phosphorescence, stimulus‐responsive

## Abstract

Stimuli‐responsive materials exhibiting exceptional room temperature phosphorescence (RTP) hold promise for emerging technologies. However, constructing such systems in a sustainable, scalable, and processable manner remains challenging. This work reports a bio‐inspired strategy to develop RTP nanofiber materials using bacterial cellulose (BC) via bio‐fermentation. The green fabrication process, high biocompatibility, non‐toxicity, and abundant hydroxyl groups make BC an ideal biopolymer for constructing durable and stimuli‐responsive RTP materials. Remarkable RTP performance is observed with long lifetimes of up to 1636.79 ms at room temperature. Moreover, moisture can repeatedly quench and activate phosphorescence in a dynamic and tunable fashion by disrupting cellulose rigidity and permeability. With capabilities for repeatable moisture‐sensitive phosphorescence, these materials are highly suitable for applications such as anti‐counterfeiting and information encryption. This pioneering bio‐derived approach provides a reliable and sustainable blueprint for constructing dynamic, scalable, and processable RTP materials beyond synthetic polymers.

## Graphical abstract

This work reports a sustainable bio‐fermentation approach to fabricate outstanding room temperature phosphorescent nanofiber membranes, overcoming the challenges associated with physical doping and chemical synthesis. By incorporating indolocarbazole derivatives into the culture medium, the rigid cellulose chains facilitate effective triplet emission, demonstrating a significant phosphorescence lifetime of 1636.79 ms and an afterglow persists for up to 10 s at room temperature.

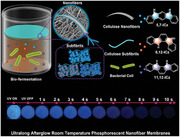

## 1 Introduction

Materials exhibiting room temperature phosphorescence (RTP) have attracted significant research interests in recent years due to their ability to emit persistent luminescence without continuous excitation, hence allowing applications in sensing,^[^ ^1^ ^]^ information security,^[^ ^2^ ^]^ anti‐counterfeiting,^[^ ^3^ ^]^ and lifetime bioimaging.^[^ ^4^ ^]^ The inorganic and organometallic RTP materials heavily rely on rare earth metals, which present challenging issues in high cost, potential toxicity, and instability in aqueous environments.^[^ ^5^ ^]^ On the contrary, organic RTP materials^[^ ^6^ ^]^ enjoy advantages such as non‐toxicity, low cost, feasible functionalization, and color tunability.^[^ ^7^ ^]^ Restriction of molecular motion is a prerequisite to achieving efficient triplet state emission. Therefore, RTP phenomena in organic small molecules are often observed in the crystalline states, which leads to poor processability and hinders their practical applications.^[^ ^8^ ^]^

Alternatively, polymer‐based organic RTP materials can provide rigid environments for long‐lived triplet excitons while maintaining good stretchability and processability.^[^ ^9^ ^]^ So far, the primary strategy focuses on physical doping, where phosphorescent dopants are blended with rigid synthetic polymers, such as poly(methyl methacrylate) (PMMA) and water‐soluble poly(vinyl alcohol) (PVA).^[^ ^10^ ^]^ Non‐covalent interactions (hydrogen bonding, dispersion forces, and electrostatic forces) between the matrix and RTP luminogens restrict the molecular motion, thus achieving efficient phosphorescence.^[^ ^11^ ^]^ However, phase separation and non‐degradability of such synthetic polymer‐based RTP materials have compromised their long‐term real‐world applications. An alternative strategy to address the phase‐separation challenge is to chemically integrate the RTP luminogens into the polymer, resulting in homogeneous polymeric RTP materials.^[^ ^12^ ^]^ However, research on these RTP materials is limited, mainly due to the challenges in multi‐step synthesis and the high cost compared to the physically doped approach. Therefore, developing sustainable, scalable, and reliable strategies for efficient polymer‐based organic RTP materials is highly desirable.

Bacterial cellulose (BC) is a type of fibrous biopolymer fabricated through the metabolic process of bacteria.^[^ ^13^ ^]^ BC biosynthesis involves the secretion of glucan chains by bacterial cells, which are arranged in a regular row along the longitudinal axis. After secretion, these chains come together to form subfibrils, with ≈10 to 15 chains associating with each other. These subfibrils then aggregate side by side, resulting in the formation of the cellulose nanofiber.^[^ ^14^ ^]^ As a distinctive kind of biosynthesized fibrous nanocellulose, BC has attracted considerable attention as a scaffold for functional materials due to its hierarchical structure, exceptional mechanical and thermal stability, as well as good biocompatibility.^[^ ^15^ ^]^ The bio‐fermentation process for BC production is simple, controllable, and environmentally friendly, offering a robust choice for green material preparation.^[^ ^16^ ^]^ Compared with commonly used artificial PMMA and PVA, BC exhibited good biocompatibility, flexibility, and high stability even under high humidity conditions. Besides, the abundant hydroxyl groups, high crystallinity, and rigidity of BC can significantly facilitate the phosphorescence emission,^[^ ^17^ ^]^ achieving efficient RTP performance.^[^ ^18^ ^]^

Inspired by the unique advantages of BC, we herein utilized biosynthesized BC as the matrix to construct polymer‐based organic RTP materials. By directly introducing indolocarbazole (ICz) derivatives as the phosphorescent dopants into the bacterial culture medium, BC‐based RTP nanofiber membranes were developed where the rigid cellulose chains could effectively encapsulate the RTP luminogens to enable persistent triplet emission. This approach offered a scalable and eco‐friendly solution for fabricating RTP materials, addressing the limitations commonly associated with traditional physical doping and chemical synthesis methods. Remarkable RTP performance is observed with long lifetimes of up to 1636.79 ms at room temperature. Intriguingly, the RTP intensity decreased by disrupting the cellulose crystallinity with moisture, thus achieving unique dynamic and reversible phosphorescence for anti‐counterfeiting and information encryption. This sustainable bio‐fabrication approach provides new opportunities to construct efficient RTP materials and solves issues of phase separation and hazardous synthesis.

## 2 Results and Discussion

**Figure 1:**
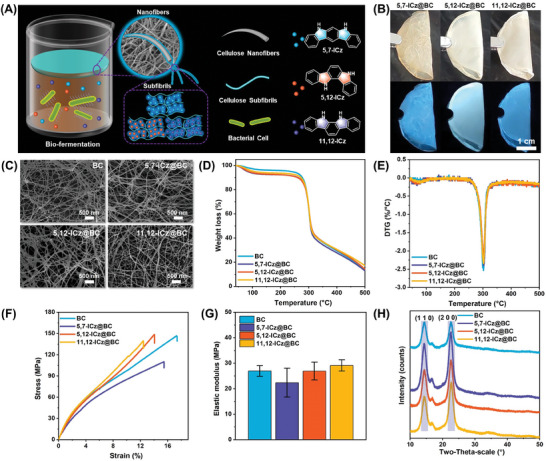
Design and Characterizations of ICz@BC: A) Schematic illustration of ICz@BC preparation and ICz molecules (**5,7‐ICz**, **5,12‐ICz**, and **11,12‐ICz**). B) Photographs of folded ICz@BC samples under daylight and 365 nm UV irradiation. C) Scanning electron microscopy (SEM) images, D) thermal gravimetric (TG) analysis, E) derivative thermogravimetry (DTG) analysis, F) tensile stress–strain curves, G) elastic modulus values (*n* = 3; means ± SD), and H) XRD spectra of BC, **5,7‐ICz@BC**, **5,12‐ICz@BC**, and **11,12‐ICz@BC**.

Three ICz isomers, namely 5,7‐dihydroindolo[2, 3‐b]carbazole (**5,7‐ICz**), 5,12‐dihydroindolo[3,2‐a]carbazole (**5,12‐ICz**), and 11,12‐dihydroindolo[2,3‐a]carbazole (**11,12‐ICz**), were selected as RTP luminogens and purified according to our previous report (**Figure** **1**A).^[^ ^11b^ ^]^ The BC was fabricated via bio‐fermentation (Figure S1, Supporting Information). Different concentrations (10, 50, 100, and 200 µm) of ICz isomers (**5,7‐ICz**, **5,12‐ICz**, and **11,12‐ICz**) were co‐cultured with bacterial strains of *Komagataeibacter xylinus* (ATCC 10245) in the culture medium and incubated for 7 days at 30 °C. The materials obtained from the above co‐culture process were termed ICz@BC. A series of uniform ICz@BC and BC pellicles were generated at the liquid‐gas interface (Figures S2A and E–P and S3, Supporting Information) with no noticeable difference between them. Afterward, the BC hydrogels were collected and washed with sodium hydroxide solution to remove the unwanted remaining microbial cells. Finally, ICz@BC membranes with thicknesses from 44.67 to 65.67 µm were obtained through vacuum drying (Table S1, Supporting Information). Notably, no BC membrane could be formed in parallel experiments with commercially available inorganic phosphorescent powders. These results indicated that the organic ICz isomers were biocompatible with bacterial metabolism compared to inorganic RTP materials.

UV–vis spectra in Figure S4 (Supporting Information) revealed that new absorbance peaks were detected in the ICz@BC membranes compared to pure BC solution. X‐ray photoelectron spectroscopy (XPS) measurements exhibited the peak of N element for ICz@BCs samples, confirming the presence of ICz molecules in the membranes (Figure S5, Supporting Information). Hence, the UV–vis and XPS jointly supported the successful integration of ICz molecules into the biopolymer matrix. The obtained ICz@BC membranes exhibited excellent flexibility and displayed vibrant blue emission under UV excitation (Figure 1B), further supporting the presence of ICz molecules within the BC membranes. SEM images were used to study the nanostructures of both the BC and ICz@BC membranes. All the membranes had porous networks with a nanofiber diameter range from 18 to 42 nm (Figure S6A, Supporting Information). At the nanoscale level, no significant differences were observed between pristine BC and ICz@BC (Figure 1C; Figure S6B, Supporting Information), suggesting that the incorporation of ICz isomers did not affect the nanostructures of the BC membranes. In contrast, ICz/BC samples with physically doped ICz isomers displayed different dense nanofiber networks without apparent porous structures (Figure S7, Supporting Information).

TG and DTG analyses were conducted on both the pristine BC and ICz@BC samples to study their thermal behaviors (Figure 1D,E). The weight loss curves of pristine BC revealed two stages of thermal decomposition. The initial stage occurred between 50 and 100 °C with minor weight loss due to removing absorbed water content in cellulose.^[^ ^19^ ^]^ The fast pyrolysis between 270 and 320 °C exhibited a significant weight loss, indicating the decomposition of the cellulose skeleton.^[^ ^20^ ^]^ Notably, the TG and DTG curves for the three ICz@BC materials exhibited similar patterns to those of pristine BC. Therefore, the introduction of ICz isomers had no significant impact on the thermal behavior of BC materials.

The mechanical properties of pristine BC, **5,7‐ICz@BC**, **5,12‐ICz@BC**, and **11,12‐ICz@BC** were also evaluated. The stress–strain curves revealed that the strains at the break for these materials were ≈17.29%, 15.43%, 14.05%, and 12.41%, respectively. The stress–strain curves of four ICz@BC materials exhibited a consistent overall trend. In the initial stage, the slope of the stress–strain curve was relatively steep, indicating a linear relationship between stress and strain, where the material was undergoing elastic deformation with a high modulus. As the stretching continued, the material underwent plastic deformation, and the slope of the stress–strain curve decreased. When the stress reached the material's ultimate strength, the material fractured without further increasing the strain. At this point, the stress–strain curve exhibited a precipitous drop. Additionally, the elastic modulus values of all materials exceeded 22 MPa (Figure 1G), with no noticeable differences in the mechanical properties among these materials. These results indicated that the incorporation of ICz isomers did not significantly affect the mechanical properties of the resulting membranes, maintaining a high level of comparable strength to pristine BC. The combination of thermal and mechanical experiments proved that the ICz@BC membranes possessed remarkable thermal stability (withstanding temperatures up to 270 °C) and high tensile strengths, making them suitable for various real‐world applications.

X‐ray diffraction (XRD) spectra were collected to analyze the crystallinity of the ICz@BC membranes, revealing two characteristic absorption peaks at ≈14.28° and 22.67° (Figure 1H). These peaks corresponded to the (110) and (200) planes of the unchanged cellulose crystalline structure, respectively.^[^ ^21^ ^]^ Therefore, the incorporation of organic ICz isomers did not have a noticeable influence on the crystal structure of BC.

**Figure 2:**
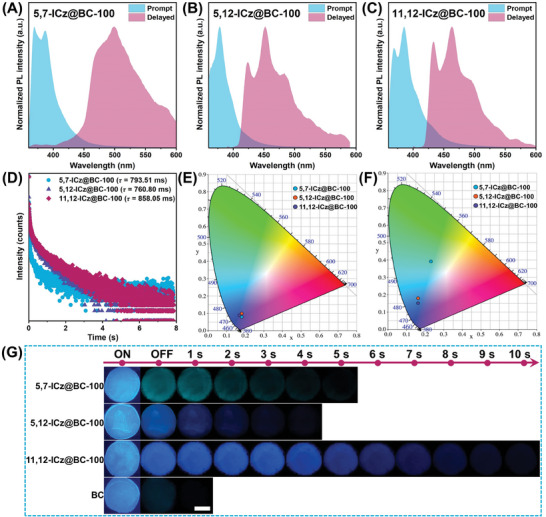
Photophysical properties of ICz@BC samples at room temperature: A–C) Prompt and delayed photoluminescence (PL) spectra. Excitation: 306, 356, and 328 nm. D) Time‐resolved phosphorescence decay curves. E,F) CIE 1931 coordinates of steady‐state PL and afterglow emission. G) Luminescence photographs before and after removing the 365 nm UV irradiation. Scale bar: 10 mm.

When ICz isomers (**5,7‐ICz**, **5,12‐ICz**, and **11,12‐ICz**) were immobilized into BC culture media with different concentrations, the phosphorescent intensity reached the strongest using 100 µm of ICz isomers (Figure S8D–F, Supporting Information). Remarkable blue or green‐colored afterglows persisted for more than 4 s (**Figure** **2**G), which was significantly longer than pure BC (Figure S9D, Supporting Information). As depicted in Figure 2A**–**C, the prompt photoluminescence (PL) spectra of ultralong RTP BC membranes (**5,7‐ICz@BC‐100**, **5,12‐ICz@BC‐100**, and **11,12‐ICz@BC‐100**) exhibited fluorescence peaks at 369, 378, and 385 nm, respectively. Their Commission Internationale de l'Eclairage (CIE) coordinates were calculated to be (0.18, 0.08), (0.18, 0.10), and (0.17, 0.09), respectively (Figure 2E). However, a significant redshift was observed between the prompt and delayed PL spectra. The phosphorescence peaks in the delayed PL spectra were located at 499, 452, and 463 nm, with the CIE coordinates as (0.23, 0.39), (0.16, 0.18), and (0.16, 0.15), respectively. Surprisingly, the lifetimes fitted from the time‐resolved decay curves of delayed emissions were remarkably long (793.51, 760.80, and 858.05 ms for **5,7‐ICz@BC‐100**, **5,12‐ICz@BC‐100**, and **11,12‐ICz@BC‐100**, as shown in **Table** **1** and Figure 2D). The CIE coordinates of the ICz@BC membranes produced from the culture medium were calculated with varying concentrations of ICz molecules (Figure S10, Supporting Information). However, no significant variations were observed, suggesting that the color of fluorescence and afterglows remained unaffected by the concentration changes.

**Table 1:** Photophysical property summary of BC and ICz@BC membranes at room temperature.

Samples	*λ*_F_ [nm]	*λ*_p_ [nm]	Φ_F_ [%]	*τ*_P_ [ms]
**BC**	400	515	2.59%	35.89
**5,7‐ICz@BC‐100**	369	499	8.14%	793.51
**5,12‐ICz@BC‐100**	378	452	13.25%	760.80
**11,12‐ICz@BC‐100**	385	463	18.39%	858.05

The presence of oxygen and water was known to affect the triplet state significantly,^[^ ^22^ ^]^ thus hindering the phosphorescence of ICz@BCs. To eliminate this influence, the samples were measured under both vacuum and hydrogel conditions (**Table** **2**; Figure S11B,C and S12, Supporting Information). As expected, longer lifetimes of 1051.40, 1172.97, and 1636.79 ms were detected under vacuum for **5,7‐ICz@BC‐100**, **5,12‐ICz@BC‐100**, and **11,12‐ICz@BC‐100**, respectively. However, the fitted lifetimes of the corresponding hydrogels were 4.44, 19.07, and 31.53 ms, respectively, which were considerably shorter compared to the dried ICz@BCs. These results suggested that oxygen and water in the air significantly influenced the RTP emissions. We conducted further analysis of the phosphorescence properties of the samples at low temperatures. The results showed that ultra‐long lifetimes of 2236.74, 2387.58, and 3113.79 ms were detected at 78 K for **5,7‐ICz@BC‐100**, **5,12‐ICz@BC‐100**, and **11,12‐ICz@BC‐100**, respectively as listed in Table 2. Additionally, it was observed that the ultra‐long afterglows persisted for more than 10 s (Figure S13, Supporting Information). The results suggested that low temperature can significantly enhance the phosphorescence emissions.

**Table 2:** Summary of phosphorescence lifetime of BC, ICz@BCs, ICz/BCs, and ICz crystals under different conditions. Water presents hydrogel materials, which was the state of the BC samples after being washed before drying. (unit: ms).

Sample	RT	Vacuum	78 K (under vacuum)	Water (hydrogel)
BC	35.89	58.77	100.30	4.14
**5,7‐ICz@BC‐100**	793.51	1051.40	2236.74	4.44
**5,12‐ICz@BC‐100**	760.80	1172.97	2387.58	19.07
**11,12‐ICz@BC‐100**	858.05	1636.79	3113.79	31.53
**5,7‐ICz/BC‐100**	30.72	81.25	847.21	32.46
**5,12‐ICz/BC‐100**	27.44	78.25	948.48	41.78
**11,12‐ICz/BC‐100**	38.64	129.94	1781.00	38.49
**5,7‐ICz** crystal	N/A	N/A	N/A	–
**5,12‐ICz** crystal	98.15	106.50	1219.87	–
**11,12‐ICz** crystal	4.47	4.37	1083.44	–

**Figure 3:**
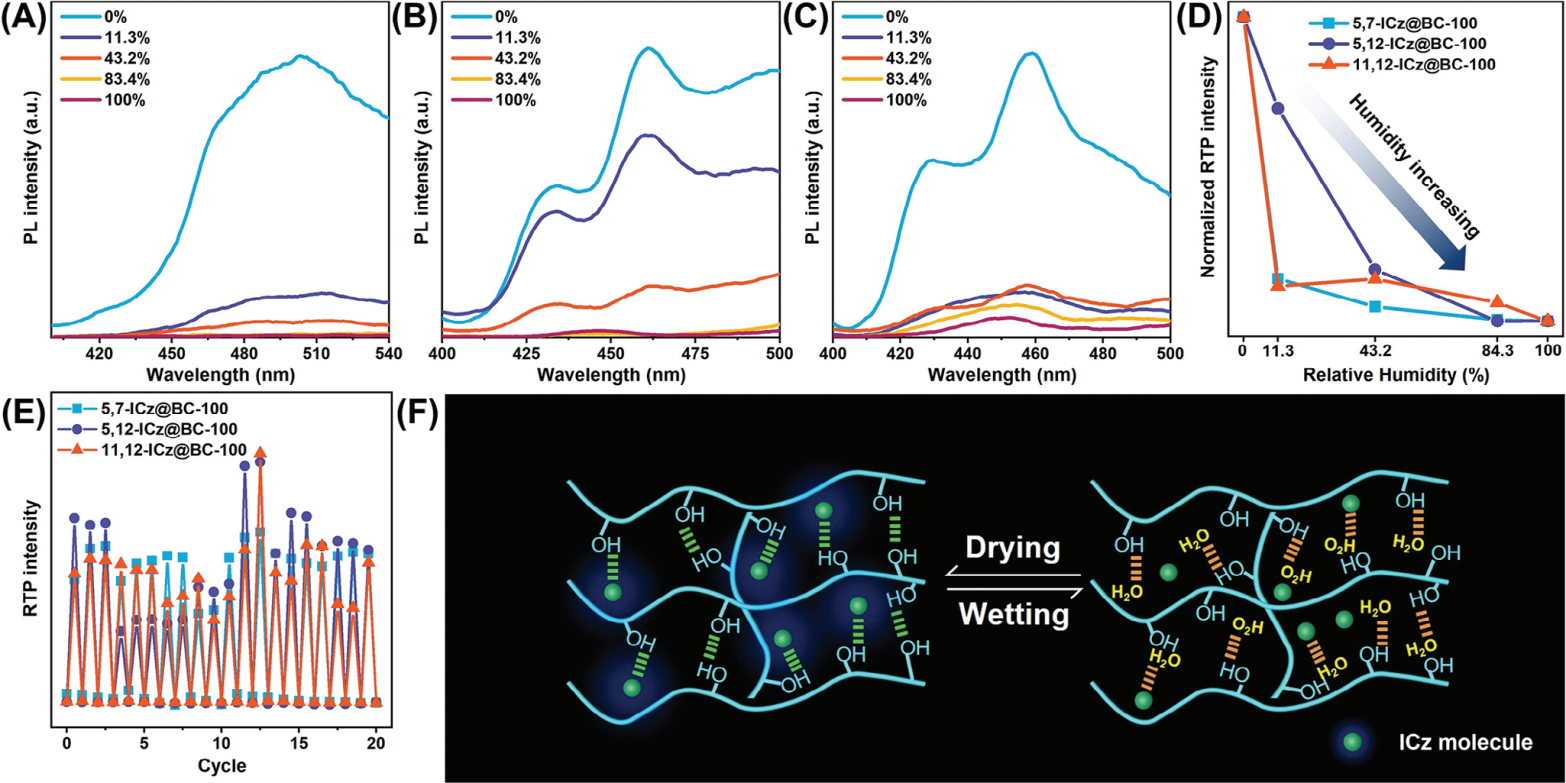
RTP properties in response to wetting/drying stimuli: Delayed PL spectra of A) **5,7‐ICz@BC‐100**, B) **5,12‐ICz@BC‐100**, and C) **11,12‐ICz@BC‐100** samples under different humidity. D) RTP intensity of the ICz@BC samples on exposure to different humidity conditions. E) Repeated water wetting/drying cycles. F) Schematic illustration of the RTP mechanism of ICz@BC in response to wetting/drying stimuli.

To further investigate the impact of moisture, we evaluate the impact of water exposure duration time on the prompt PL properties, and excellent fluorescence stability was observed (Figure S14, Supporting Information). Afterward, the RTP behaviors of **5,7‐ICz@BC‐100**, **5,12‐ICz@BC‐100**, and **11,12‐ICz@BC‐100** subjecting to alternating wetting and drying were studied. The RTP behaviors of all the ICz@BCs were responsive to humidity, showing a decrease in intensity as the ambient humidity level increased (**Figure** **3**A–D), and the RTP was restored after drying (Figure S15, Supporting Information). The wetting‐drying cycle could be repeated twenty times without obvious decay (Figure 3E). Fourier transform‐infrared spectroscopy (FT‐IR) analyses were conducted to investigate the moisture‐sensitive mechanism. A peak near 3446 cm^−1^ could be observed in the dry membrane, corresponding to the stretching vibration of water and hydroxyl groups (─OH)^[^ ^23^ ^]^ in the BC matrix (Figure S16, Supporting Information). The peak shape became broader after exposure to water vapor. Based on these findings, the moisture‐sensitive mechanism of the ICz@BCs was illustrated in Figure 3F. The water molecules disrupted the hydrogen bonding interactions, altering the cellulose's rigid environment and finally leading to RTP quenching. In contrast, water molecules were removed after drying to recover the intermolecular hydrogen bonds and RTP properties.^[^ ^12e^ ^]^ These results paved the way for potential applications in moisture‐sensitive RTP systems.

The photophysical properties of ICz@BCs provided compelling evidence for the fluorescence and phosphorescence characteristics, indicating the successful incorporation of ICz molecules. Compared with the cellulose‐based RTP materials from the literature,^[^ ^24^ ^]^ the **11,12‐ICz@BC‐100** membrane demonstrated outstanding RTP properties, exhibiting an exceptionally long lifetime of 858.05 ms (Table S2, Supporting Information). Another series of control samples, noted as ICz/BC, were prepared by mechanically crushing BC nanofibers and then physically mixing with ICz isomers. The lifetime values of **5,7‐ICz/BC‐100**, **5,12‐ICz/BC‐100**, and **11,12‐ICz/BC‐100** were 30.72, 27.44, and 38.64 ms, respectively (Table 2; Figure S17, Supporting Information), which were significantly shorter than those observed for the ICz@BCs. The difference in lifetimes between ICz@BC and ICz/BC materials might be attributed to the distinct combinations of molecules and nanofibers. During the biosynthesis of BC in the culture media,^[^ ^14^ ^]^ the fabrication of ICz@BC was interrupted by the small organic molecules, because the ICzs existed at all stages of BC formation and undoubtedly interact with glucan chains, subfibrils, and cellulose nanofibers, leading to uniform distribution inside and on the surface of nanofibers (Figure S18A, Supporting Information). The rigid environment of cellulose restricted the molecular motions within the nanofiber, facilitating the isolation of water and oxygen and boosting the RTP performance of the material. On the other hand, in the case of ICz/BC, the molecules tended to aggregate solely on the surface of the nanofibers through simple physical adhesion (Figure S18B, Supporting Information). As a result, the RTP performance of ICz/BC could not fully benefit from the rigid interior environment provided by cellulose. Additionally, the molecules had a significant chance of contacting water and oxygen, which would adversely affect the RTP performance of the material.

A comparison was conducted between the RTP emission spectra of the ICz@BC membranes and the delayed emission spectra of the ICz isomers in dilute solutions at 78 K (Figure S19, Supporting Information). A significant overlap between the two spectra was observed, indicating that the ultralong RTP performances of the ICz@BC membranes were due to the inherent single‐molecular phosphorescence of the ICz isomers, which were well‐dispersed among the cellulose chains at low concentrations. In the previous work of ICz isomers doped in a PMMA matrix,^[^ ^11b^ ^]^ the composite membranes did not exhibit detectable phosphorescence signals, because PMMA couldn't form extensive hydrogen bonding necessary for minimizing non‐radiative deactivation and promoting intersystem crossing. On the contrary, the abundant hydroxyl groups of the cellulose chains in BC could induce efficient and ultralong RTP emissions by forming multiple hydrogen bonds with the ICz molecules.

**Figure 4:**
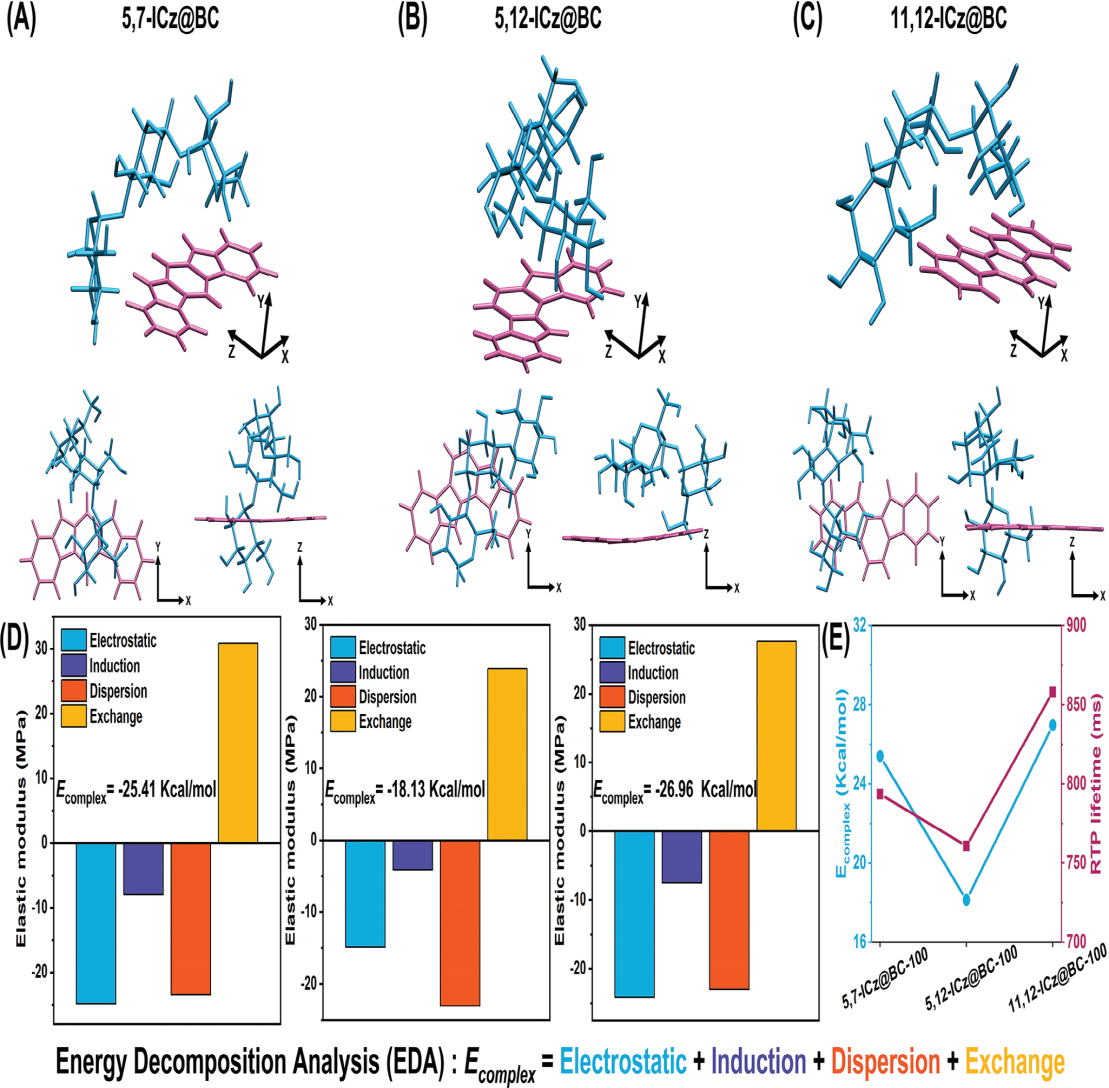
Calculated complexation energies between ICz molecule and cellulose, energy decomposing analysis diagrams, and the relationship between the complexation energy and RTP lifetime.

In order to better understand how hydrogen bonding interactions induced the ultralong RTP emission of ICz@BC systems, a quantum chemistry technique called symmetry‐adapted perturbation theory (SAPT) was used in combination with the jun‐cc‐pVDZ basis set to determine the total complexation energies between ICz isomers and cellulose.^[^ ^25^ ^]^ SAPT calculations naturally separated the total complexation energy into physical electrostatic, induction, dispersion, and exchange energy components. **Figure** **4** illustrated the total complexation energies (*E*_complex_) of **5,7‐ICz@BC**, **5,12‐ICz@BC**, and **11,12‐ICz@BC**, which were determined to be −25.41, −18.13, and −26.96 Kcal mol^−1^, respectively. The more negative *E*_complex_ value, the stronger the intermolecular interactions within the ICz@BC systems. Notably, it was found that the lifetime of RTP in the ICz@BC system tended to become longer (τ**_11,12‐ICz@BC_** > τ**_5,7‐ICz@BC_** > τ**_5,12‐ICz@BC_**) as the value of *E*_complex_ increased (Figure 4E). Therefore, stronger hydrogen bonding interactions between the ICz molecule and cellulose facilitated their bonding, ultimately enhancing the RTP properties. Furthermore, the energy decomposing analysis (EDA) method was used to analyze the intermolecular interactions between ICz isomers and cellulose qualitatively. Both electrostatic attraction and dispersion forces substantially contributed to the total complexation energy (Figure 4; Table S3, Supporting Information).

**Figure 5:**
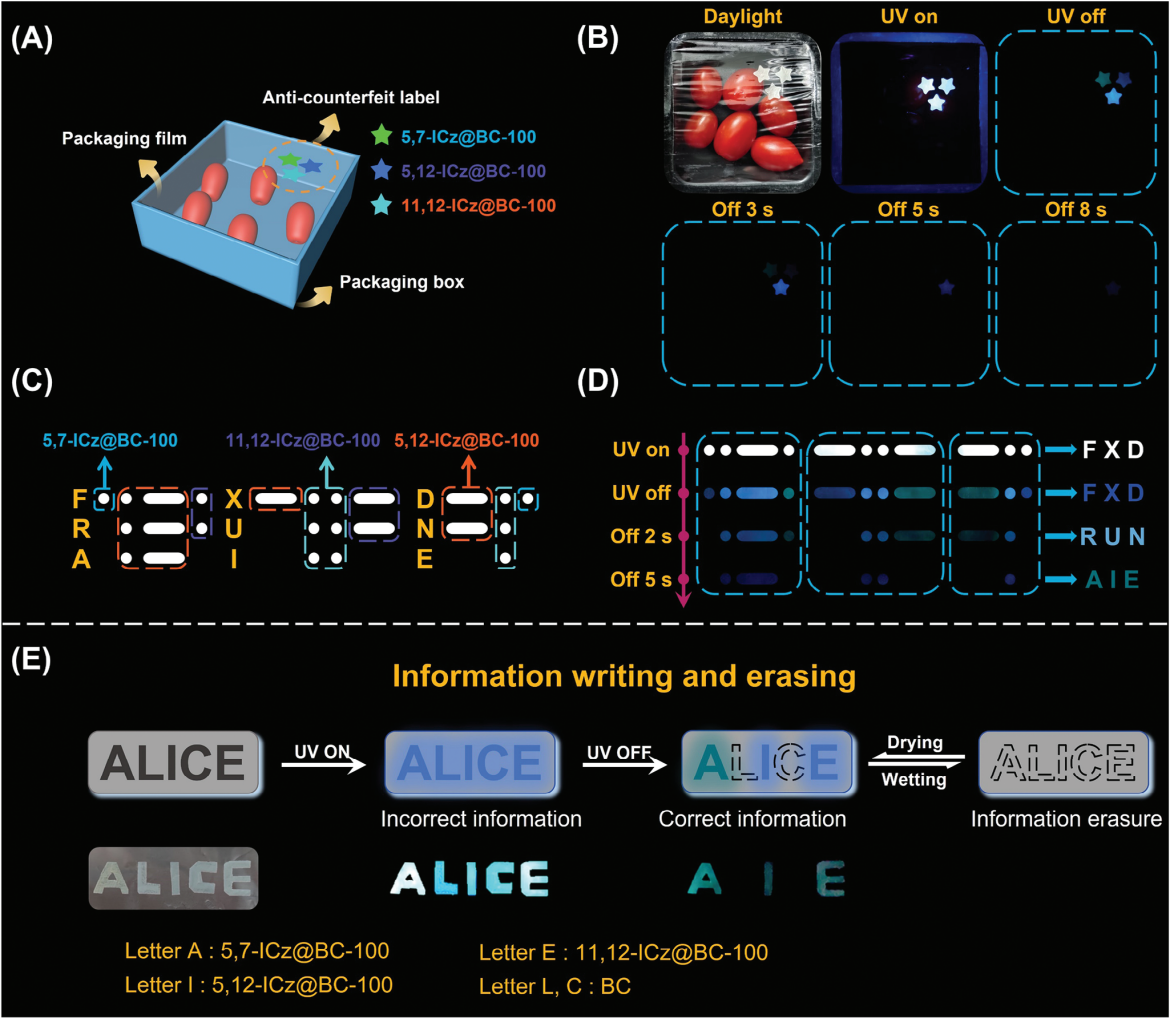
Applications of BC and RTP ICz@BC samples: A) Schematic illustration of ICz@BC membranes used in food packaging anti‐counterfeiting materials. B) Digital photographs of food packaging anti‐counterfeiting logo application. C) Schematic illustration of the Morse code message encryption pattern corresponding to different BC samples. D) Dynamic evolution photographs of the dot “•” and dash “▬“ pattern, which can be multi‐encrypted as ”AIE." E) Schematic illustration and photograph of reverse information encryption process in response to wetting/drying stimuli, which can achieve the erasure and reproduction of confidential information.

The RTP ICz@BCs had several advantages that made them useful in various applications. First, the biocompatibility and food safety made them suitable for food packaging anti‐counterfeiting. Cell toxicity experiments were performed using three ICz@BC‐200 samples to support this (Figure S21, Supporting Information), and all the cell viability exceeded 80%, supporting these materials were biocompatible. Subsequently, three white stars were made from different RTP ICz@BCs (**Figure** **5**A,B). Upon UV light excitation, a vibrant blue emissive logo became visible. This conventional mode served as the first anti‐counterfeiting level, which was easy to reproduce. However, after removing the excitation, the logo with long‐lasting afterglow was observed as the second anti‐counterfeiting level. The afterglow star numbers gradually decreased until no emission could be observed, serving as the third level of anti‐counterfeiting.

The spatial‐time‐dual‐resolved encryption functionality could also be achieved. The ICz@BCs were cut into dot “•” and dash “▬” patterns, representing the fundamental elements of the International Morse Code. After specific permutation (Figure 5C), the patterns assumed an identical optical appearance under ambient light. However, the real information “AIE” was decrypted through several steps (Figure 5D). Upon excitation by UV light, a series of the bright bluish dots “•” and dashes “▬” became visible, corresponding to the English letters “F”, “X”, and “D”. When the UV light was switched off, the letters persisted, but providing meaningless and wrong information. The pattern subsequently transformed into “R”, “U”, and “N”, which was still not the correct information. Finally, the pattern combination unveiled the accurate and vital information of “AIE”, representing “Aggregation‐Induced Emission”. Furthermore, in light of the water‐responsive RTP behavior, the encryption technology enabled both writing and erasing. As shown in Figure 5E, the word “ALICE” was prepared using **5,7‐ICz@BC‐100**, **5,12‐ICz@BC‐100**, **11,12‐ICz@BC‐100**, and BC materials. After excitation, the word “ALICE” transformed into “AIE” with long‐lasting RTP. Significantly, the “AIE” word could be erased by exposure to moisture and recovered by heating, realizing a dynamic encryption technology. These findings demonstrated the versatility and practicality of ICz@BCs in developing advanced security measures.

## 3 Conclusion

In summary, we have successfully developed a facile, scalable, and eco‐friendly strategy for constructing a series of organic RTP materials marked as ICz@BCs. These materials exhibited remarkable millisecond‐scale excited state lifetimes (858.05 ms) and up to 10 s of persistent luminescence under ambient conditions. The lifetime could reach 1636.79 ms under vacuum conditions at room temperature. The underlying principle involved utilizing cellulose chains to immobilize ICz molecules through hydrogen bonding. The cellulose matrix provided a rigid environment, stabilizing the triplet excitons and protecting them from water and oxygen. This approach offered a universal and accessible way to design highly efficient RTP materials that could be visually detected by the naked eye under ambient conditions. The varying afterglow durations of the ICz@BCs enable their applications in anti‐counterfeiting measures and multi‐level encryption patterns. Of particular interest was the unique moisture‐responsive RTP mechanism exhibited by these materials, which has allowed us to develop rewritable spatial‐time‐dual‐resolved encryption applications. Our work presented a rational and practical approach to developing environmentally sustainable RTP materials as a green alternative for artificial polymers. This research may inspire future investigations into novel persistent luminescence materials and their diverse applications.

## Conflict of Interest

The authors declare no conflict of interest.

## Author Contributions

X.N., Z.Q., and B.Z.T. conceived the idea for this work. X.N. prepared the samples, conducted the experiments, collected and analyzed the data, took the images, and wrote and revised the manuscript. J.G. and B.W. carried out the theoretical calculations. W.J.W. carried out the biotoxicity experiments. Z.D., F.G., and P.A. carried out the formal analysis. Z.Q. and B.Z.T. revised the manuscript.

## Supporting information

Supporting Information

## Data Availability

The data that support the findings of this study are available on request from the corresponding author. The data are not publicly available due to privacy or ethical restrictions.
