# Supplementary material for: Room Temperature Phosphorescent Nanofiber Membranes by Bio‐Fermentation
Source: Adv Sci (Weinh). 2024 Jul 1;11(33):2405327. doi: 10.1002/advs.202405327 (PMC11434032; doi:10.1002/advs.202405327)
Supplement: Supplementary file 1 — Supporting Information [file ADVS-11-2405327-s001.docx]

Supporting Information

**Room Temperature Phosphorescent Nanofiber Membranes by Bio-fermentation**

Xiaolin Nie,^1^ Junyi Gong,^1^ Zeyang Ding,^1^ Bo Wu,^1^ Wen-Jin Wang,^1^ Feng Gao,^1^ Guoqing Zhang,^4^ Parvej Alam,^1^ Yu Xiong,^3^ Zheng Zhao,^1^ Zijie Qiu,^1,^* Ben Zhong Tang^1,2,^*

^1^ School of Science and Engineering, Shenzhen Institute of Aggregate Science and Technology, Clinical Translational Research Center of Aggregation-Induced Emission, The Second Affiliated Hospital, The Chinese University of Hong Kong, (CUHK-Shenzhen), Shenzhen, Guangdong, 518172, P.R. China

^2^ Department of Chemistry, Hong Kong Branch of Chinese National Engineering Research Center for Tissue Restoration and Reconstruction, The Hong Kong University of Science and Technology, Kowloon, Hong Kong, 999077, P.R. China

^3^ Center for AIE Research, Shenzhen Key Laboratory of Polymer Science and Technology, Guangdong Research Center for Interfacial Engineering of Functional Materials, College of Materials Science and Engineering, Shenzhen University, Shenzhen, 518061, P.R. China

^4^ Hefei National Research Center for Physical Sciences at the Microscale, University of Science and Technology of China, Hefei, 230026, P.R. China

*Corresponding author:

zijieqiu@cuhk.edu.cn (Z. Qiu)

tangbenz@cuhk.edu.cn (B. Z. Tang)

**1. General methods**

**1.1 Fabrication of BC samples**

**Bacterial strain and culture medium:**

*Komagataeibacter xylinus* (ATCC 10245) was used for BC fermentation and fabrication.^[1]^ Culture media consisted of tryptone (3 g/L), yeast extract (5 g/L) and D-mannitol (25 g/L). The culture media was sterilized at 121 ℃ for 30 min before fermentation.

**Microbial fermentation:**

*Komagataeibacter xylinus* (ATCC 10245) was inoculated into the prepared liquid medium and cultured at 30 °C for seven days. The obtained pristine BC samples were treated with a sodium hydroxide solution (1%, w/v) overnight at 60 °C and then thoroughly washed with deionized water until the pH reached 7.0 and no fluorescence was detected in the wash water. After removing all the unwanted sugar, the BC samples were dried at 60 °C in a constant-temperature drying oven.

**Fabrication of** **ICz@BC:**

A series of ICz@BC samples were fabricated in the culture media supplemented with **5,7-ICz**, **5,12-ICz**, and **11,12-ICz** indolocarbazole isomers (10, 50, 100, and 200 μM). The fermentation and washing were performed in the same manner as the neat BC preparation.

**Fabrication of ICz/BC:**

Indolocarbazole isomers (**5,7-ICz**, **5,12-ICz**, and **11,12-ICz**) were dissolved in DMSO (100 μM), and then BC suspension, which was smashed by a homogenizer and ultrasonication, was added. After the uniform suspension was vacuum-filtered, an ICz/BC nanofiber membrane was formed. The resultant ICz/BC membranes were dried at 60 °C in a constant-temperature drying oven.

**MTT assay**:

MTT assay was performed to evaluate the biocompatibility of ICz@BC-200 samples. Briefly, LO2 cells were seeded in a 96-well plate and treated with the sterilized ICz@BC-200 samples. After incubation for 24 h, the medium was removed, followed by adding 200 fresh culture media containing MTT (0.25 mg mL^-1^). The plate was further incubated for another 4 h at 37 ^o^C in a humidified environment containing 5% CO_2_. Then, the culture media was removed, and 0.15 mL DMSO was added to each well. After 10 min incubation in a shaker, 0.1 mL of the solution was transferred to another 96-well plate. A microplate spectrophotometer recorded the absorbance at 595 nm.

**1.2 Instruments**

Scanning electron microscopy observations (SEM) of the nanofiber membranes were imaged on a TESCAN SOLARIS GMH. Thermogravimetric analysis (TGA) was conducted on a Mettler Toledo TGA2 instrument. Mechanical tension experiments were applied on an Instron universal tester 68TM-5. Powder X-ray diffraction (XRD) experiments were tested on a Rigaku Ultima IV diffractometer with Cu Kα radiation. Photoluminescence spectra (PL) were recorded on an Edinburgh FLS 1000 Instrument. UV-vis absorption spectra were recorded on a Shimadzu UV3600 spectrometer. X-ray photoelectron spectra (XPS) analysis was performed on Axis Supra+. Fourier transform-infrared spectroscopy (FT-IR) was recorded on a Nicolet Nexus spectrometer (Nicolet iS 10).^1^H and ^13^C NMR spectra were measured on a Bruker AVANCE NEO 500 NMR spectrometer using DMSO-*d_6_* as solvent.

**1.3 Theoretical Calculation**

The total complex energy was calculated using a symmetry-adapted perturbation theory (SAPT) under SAPT0/jun-cc-pVDZ level of corresponding compounds with a segment from the cellulose chain in the Psi4 software package.^[2]^ The total complexation energy was decomposed into electrostatic, induction, dispersion, and repulsion energies by energy decomposition analysis (EDA).

**1.4 Statistical Analysis**

Results are expressed as the mean ± SD. All the statistical calculations were performed using the Origin 2018 edition (OriginLab Corporation).

**2. Supporting Figures and Tables**


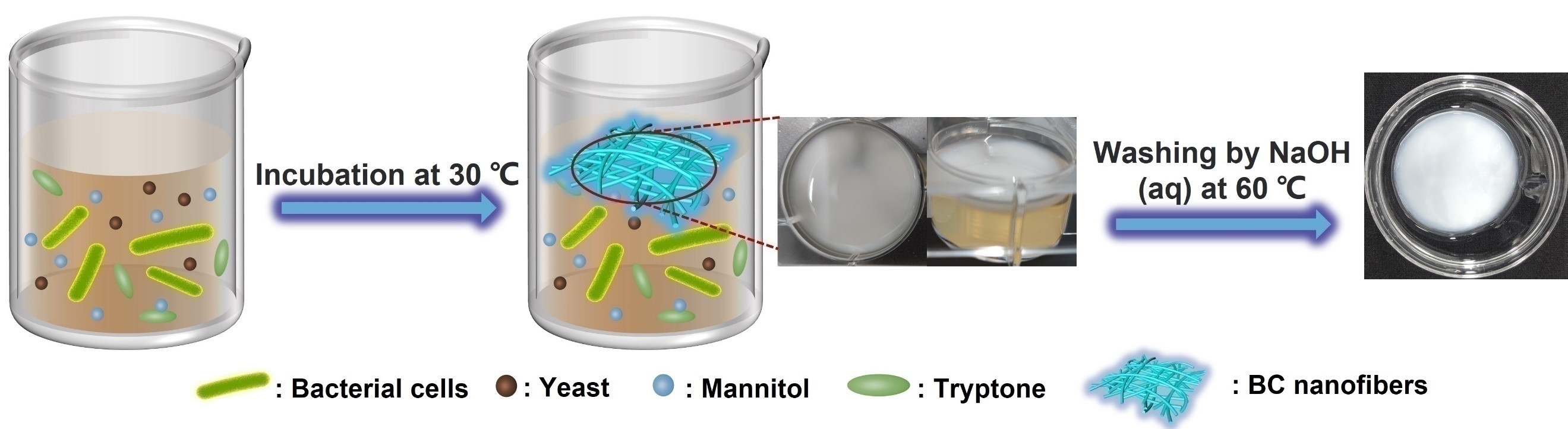


**Figure S1.** Fabrication of bacterial cellulose (BC).


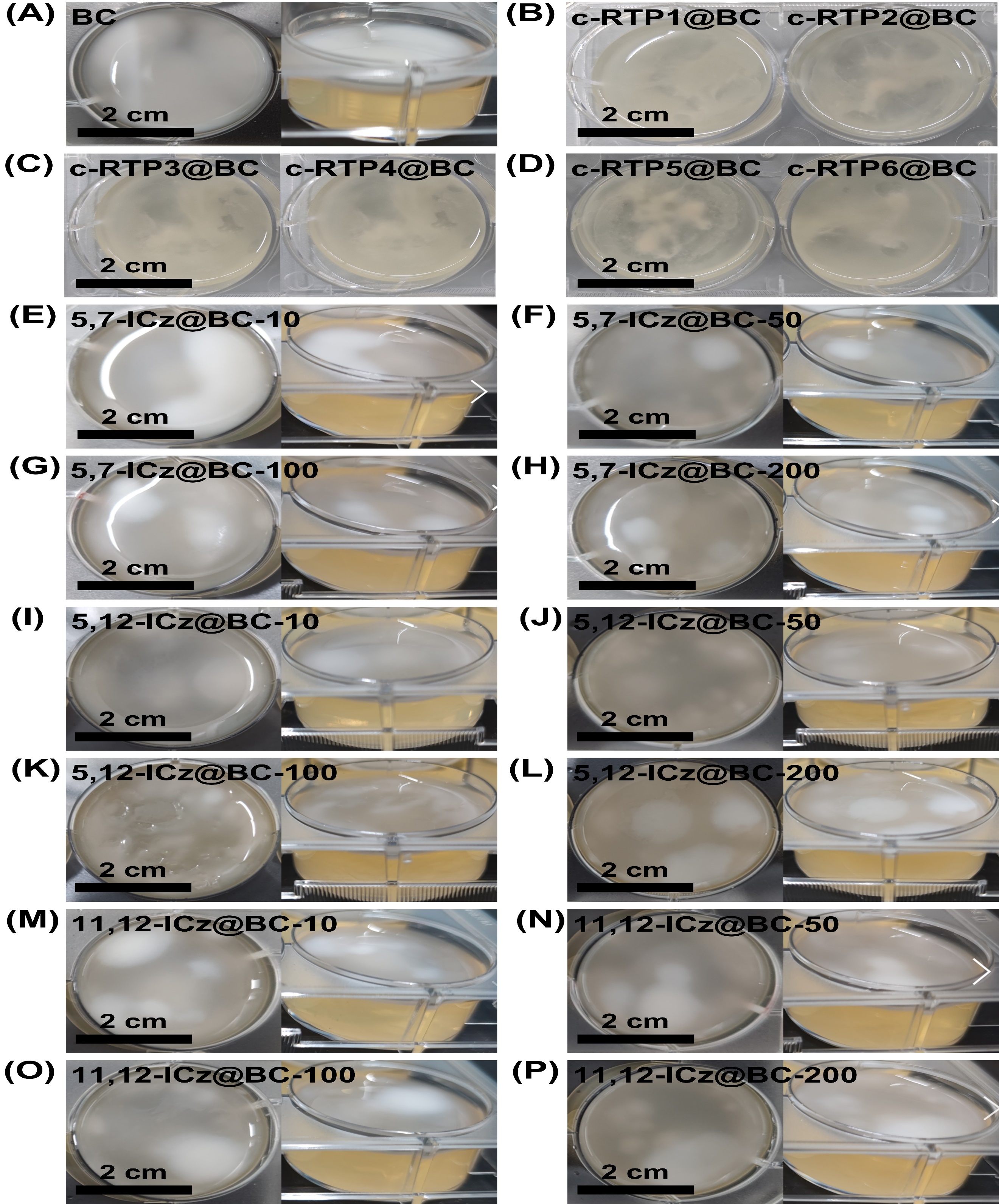


**Figure S2.** A series of BC samples in fermentation: (A) pristine BC. (B-D) BC fermentation with commercial inorganic RTP materials (c-RTP material No. 1-6). (E-P) BC fermentation supplemented with different concentrations of organic ICz molecules.


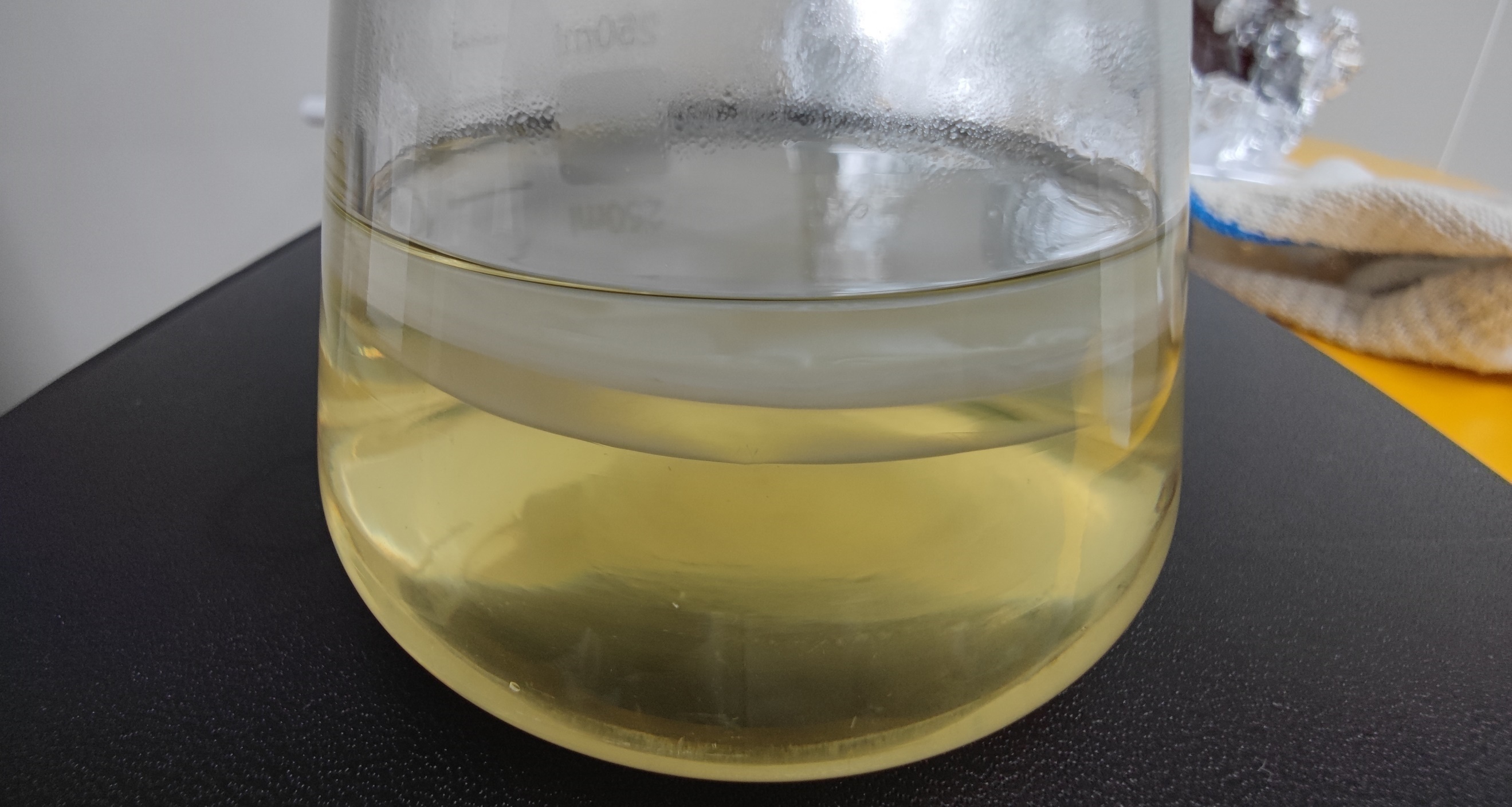


**Figure S3.** BC fermentation in a large-size fermentation bottle.


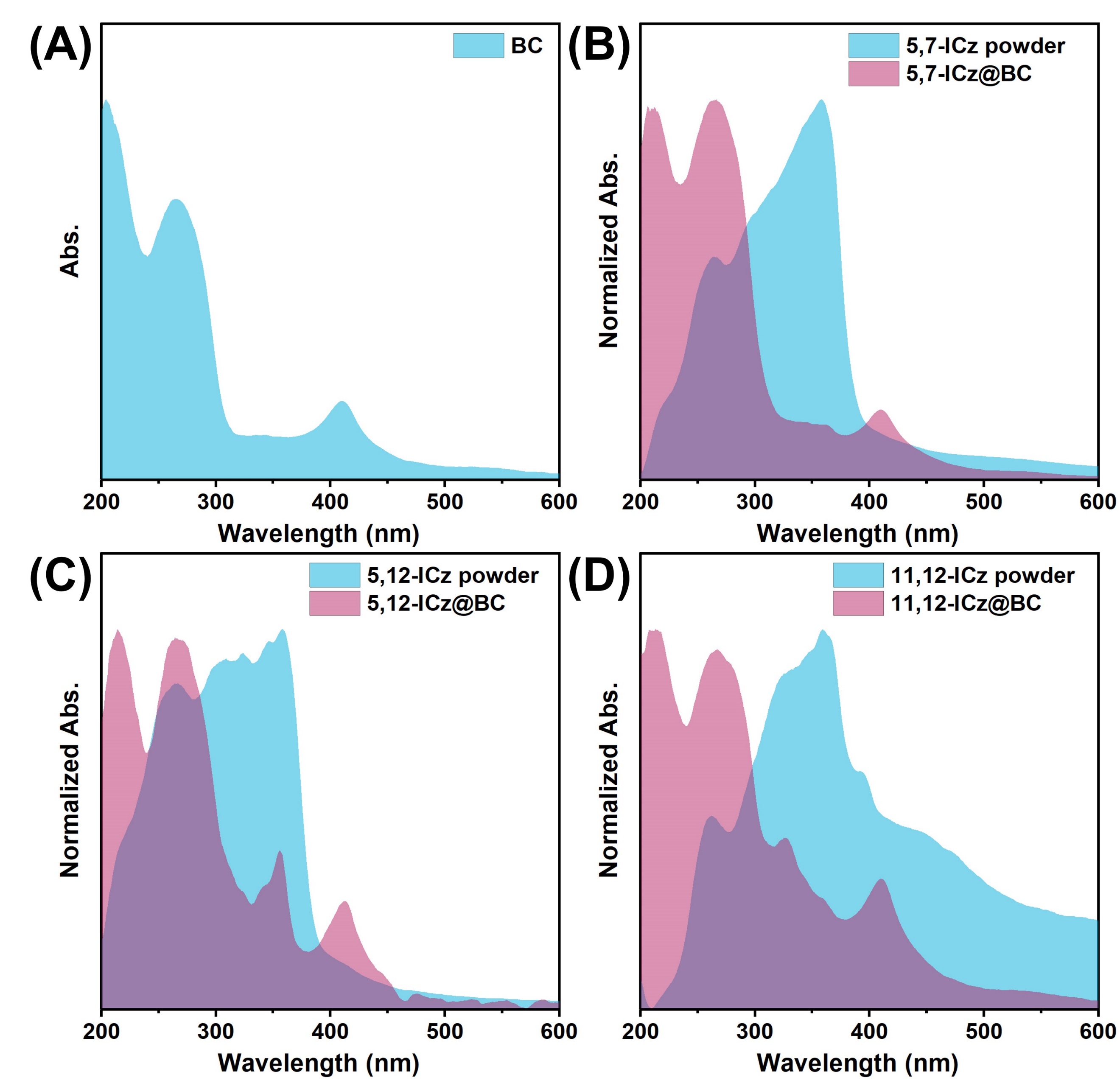


**Figure S4.** UV-vis spectra of BC and ICz@BC membranes.


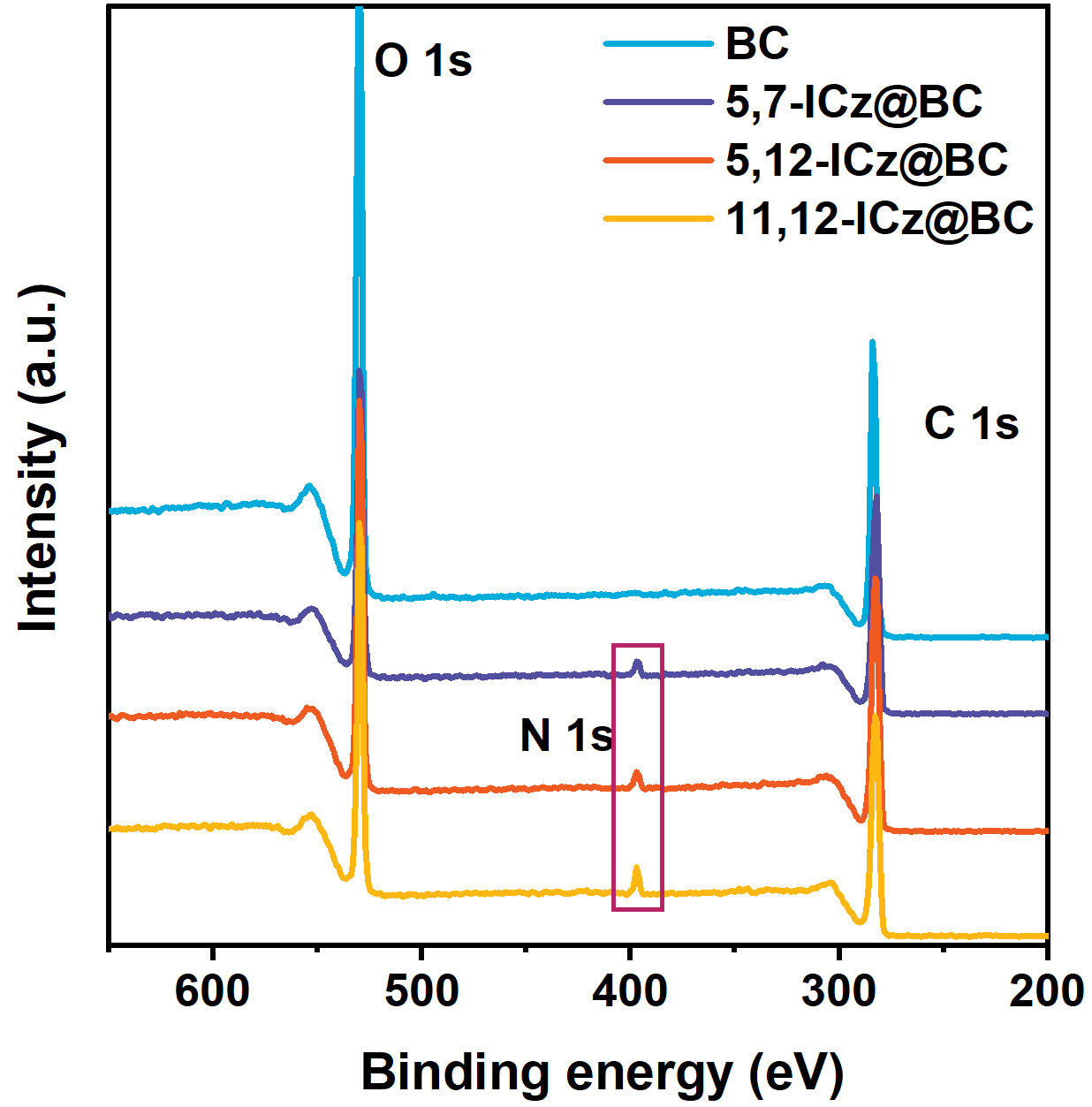


**Figure S5.** XPS spectra of BC and ICz@BC membranes.


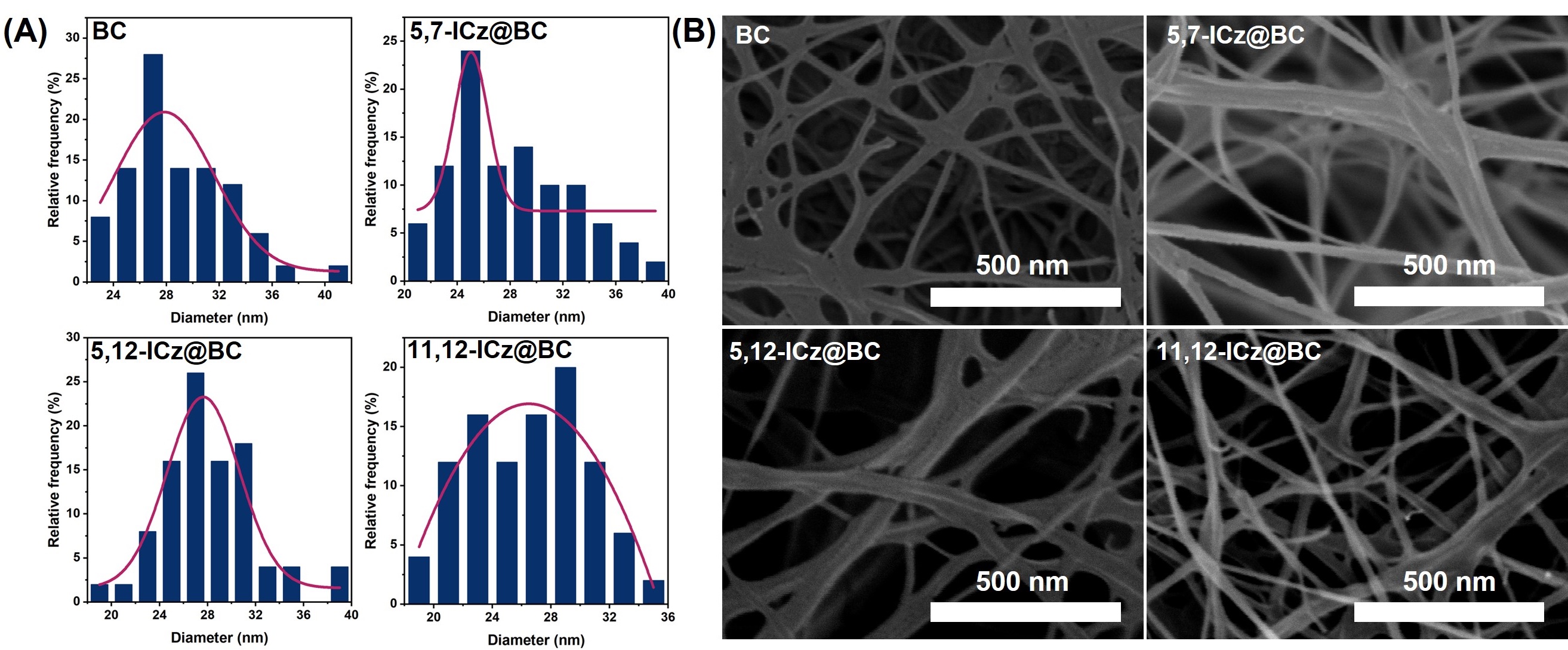


**Figure S6.** (A) Nanofiber diameter distribution from Figure 1C. (B) Enlarged SEM images of BC and ICz@BC samples.


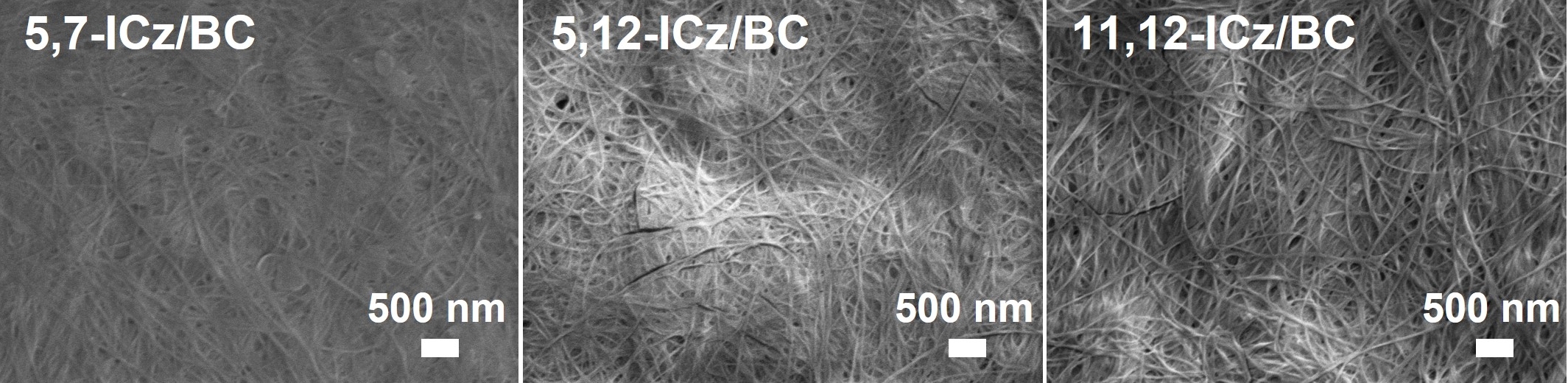


**Figure S7.** SEM images of ICz/BC samples.


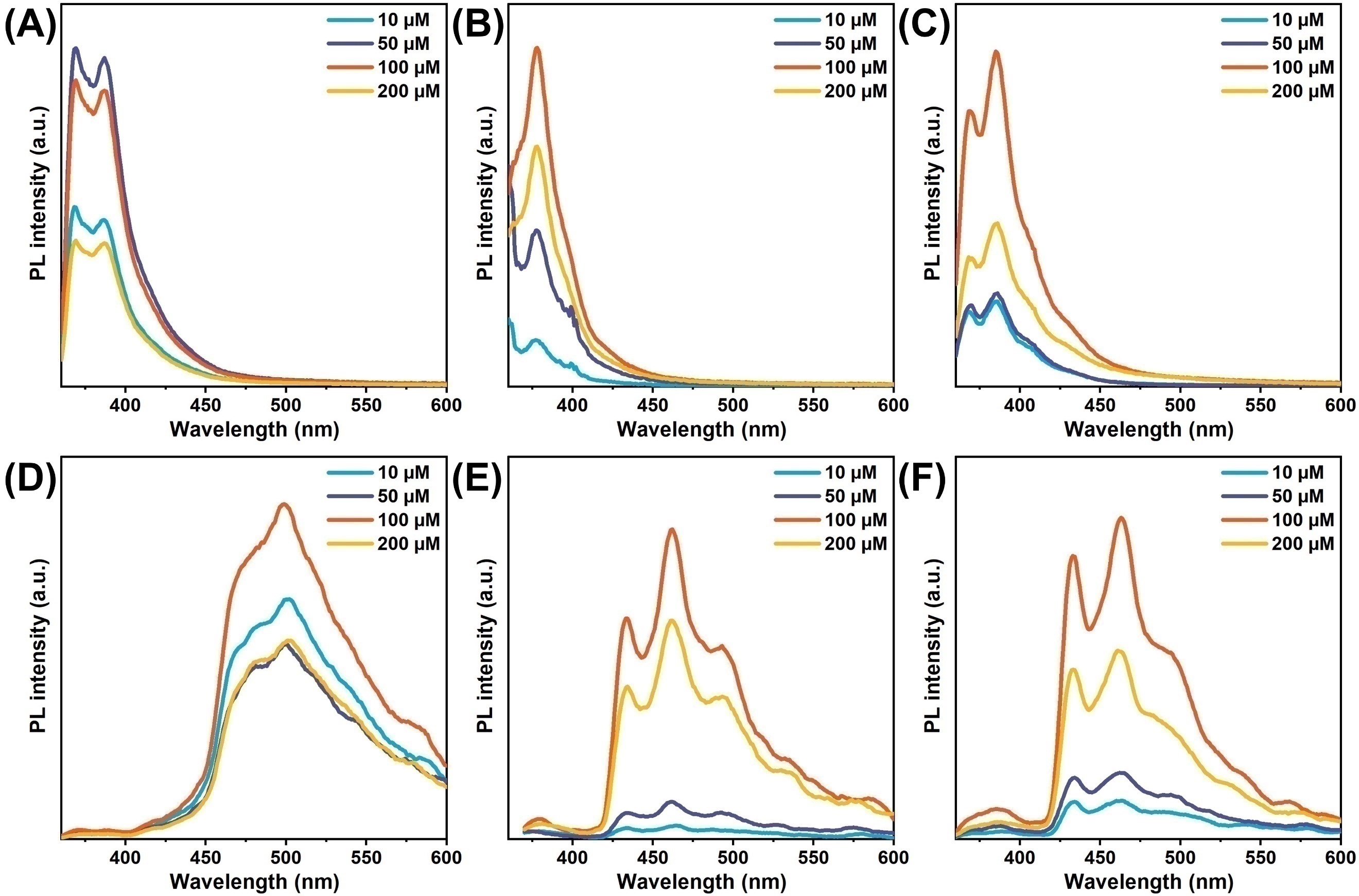


**Figure S8.** Concentration dependence of prompt and delayed PL spectra of samples (A, D) **5,7-ICz@BC**, (B, E) **5,12-ICz@BC**, and (C, F) **11,12-****ICz@BC** at room temperature.


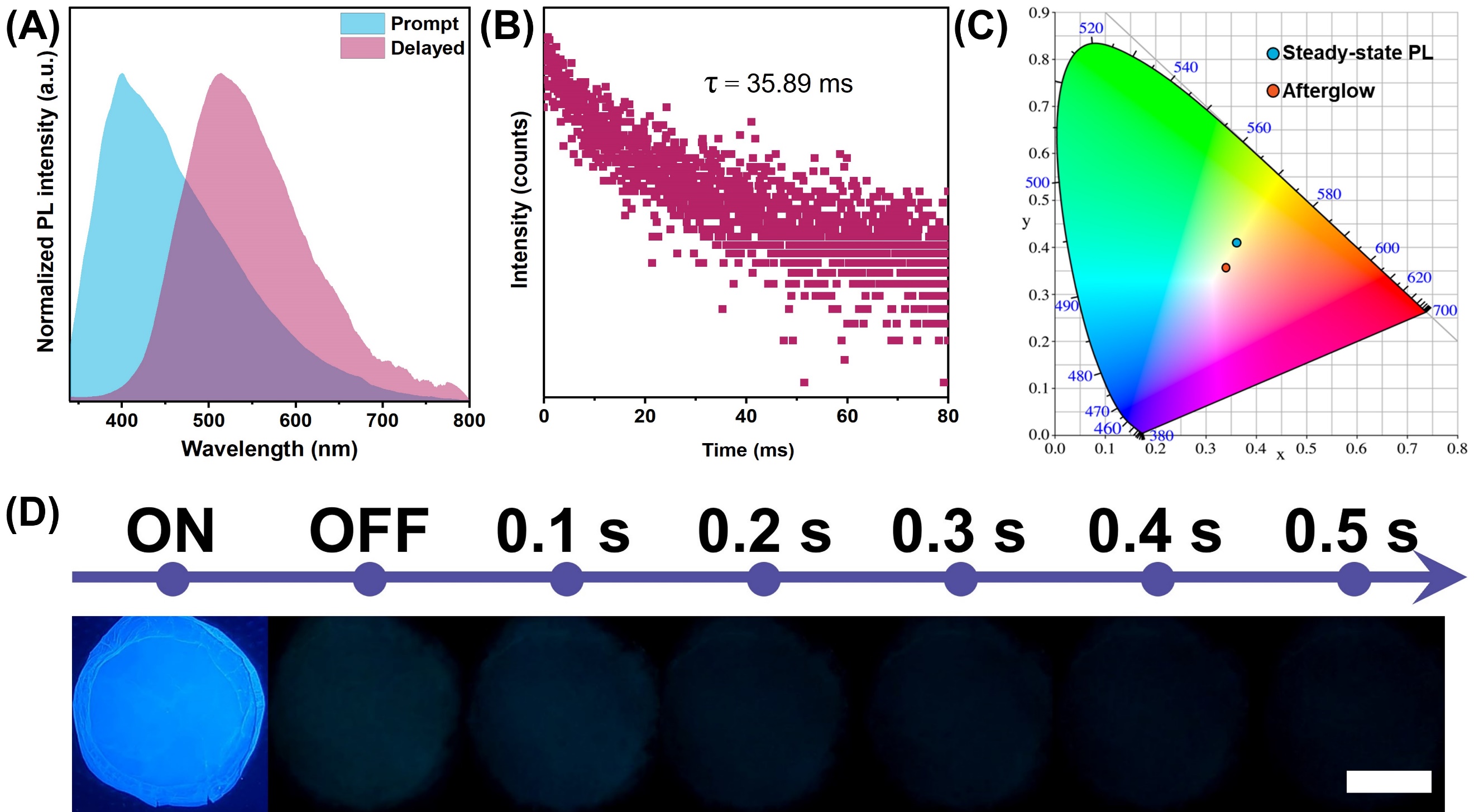


**Figure S9.** **Photophysical properties of pristine BC at room temperature:** (A) Prompt and delayed PL spectra. (B) Time-resolved phosphorescent decay curves. (C) CIE 1931 coordinates of steady-state PL and afterglow emission. (D) Luminescence photographs under and after removing the 365 nm UV irradiation. Scale bar: 10 mm.


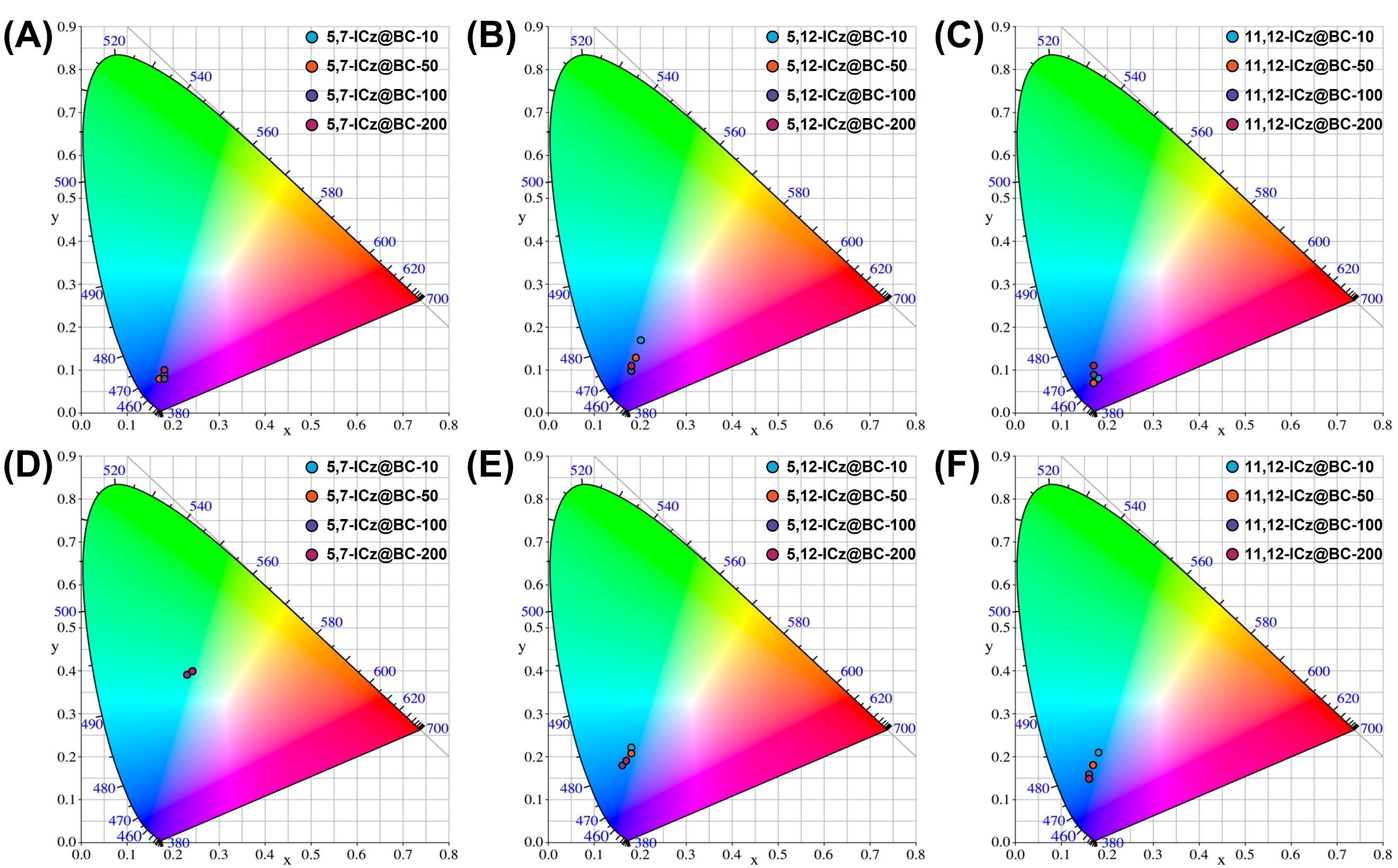


**Figure S10.** CIE 1931 coordinates of (A-C) steady-state PL and (D-F) afterglow emission of the ICz@BC membranes as a function of concentration.


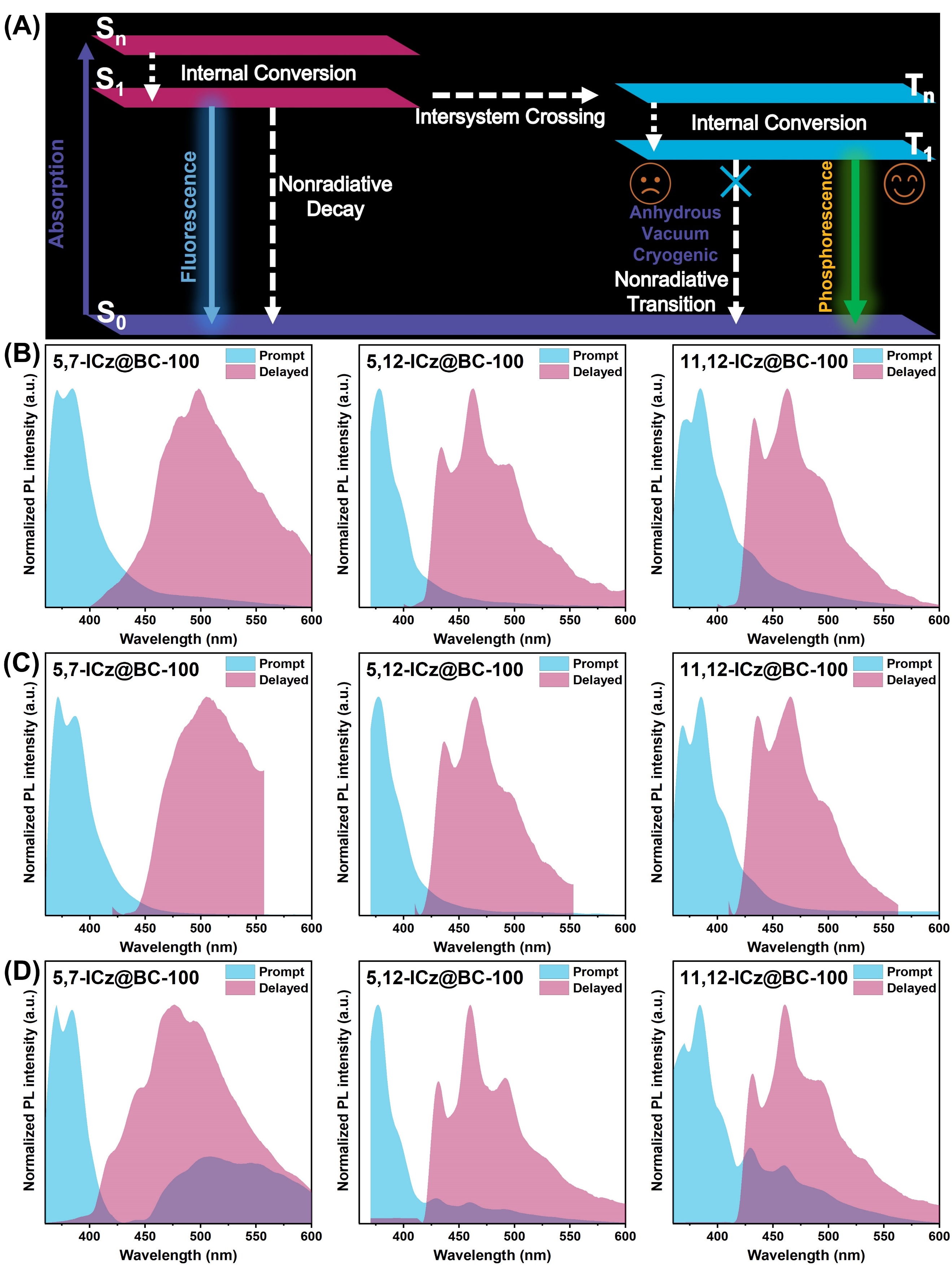


**Figure S11.** (A) Illustration of quenching effect on RTP emission. Prompt and delayed PL spectra of BC samples under (B) vacuum, (C) hydrogel, and (D) 78 K under vacuum.


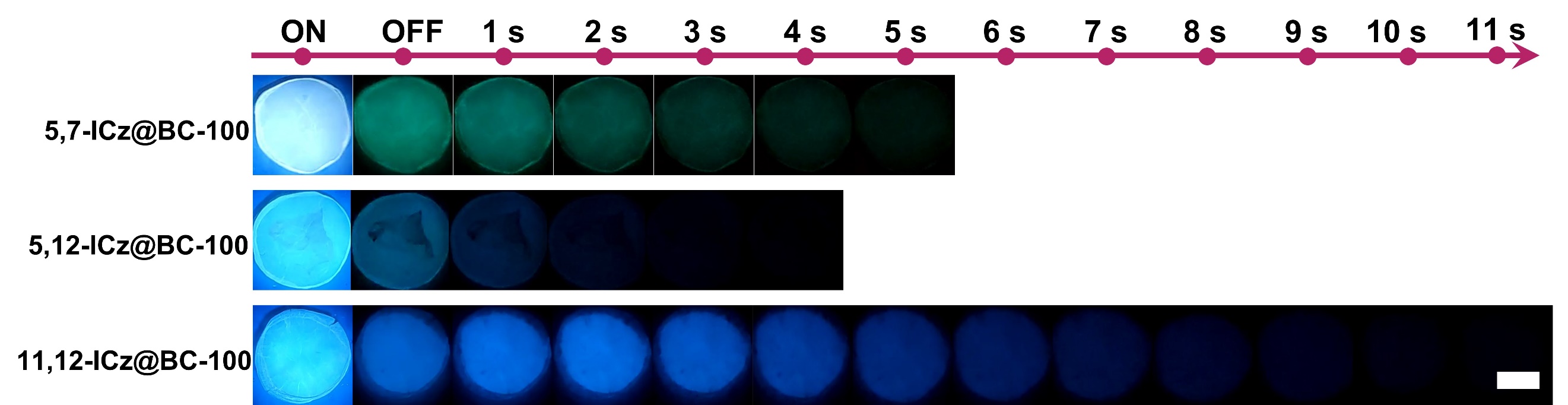


**Figure S12.** Luminescence photographs under and after removing the 365 nm UV irradiation in a vacuum. Scale bar: 10 mm.


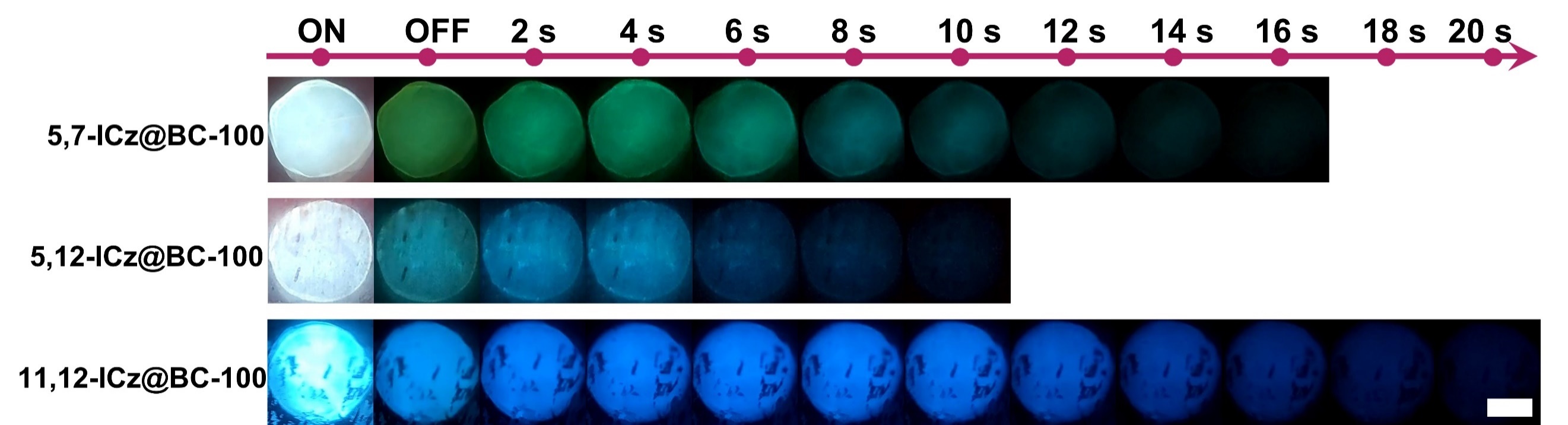


**Figure S13.** Luminescence photographs under and after removing the 365 nm UV irradiation at 78 K. Scale bar: 10 mm.


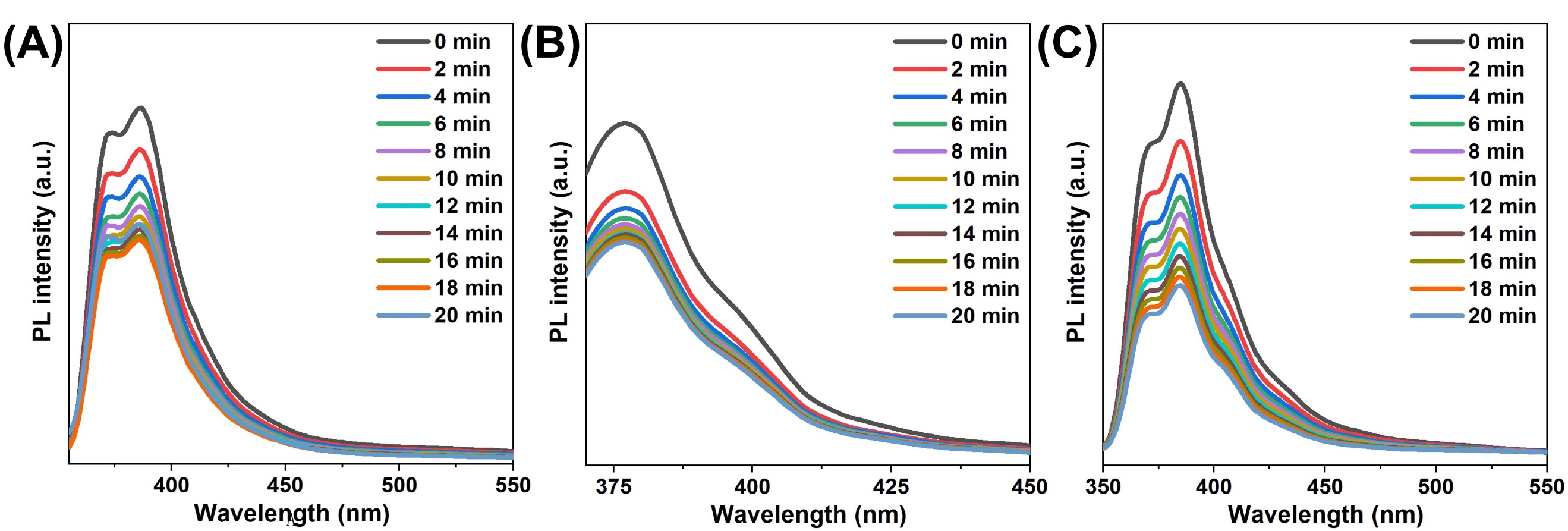


**Figure S14.** Prompt PL spectra of (A) **5,7-ICz@BC-100**, (B) **5,12-ICz@BC-100**, and (C) **11,12-ICz@BC-100** with increasing exposure time to moisture.


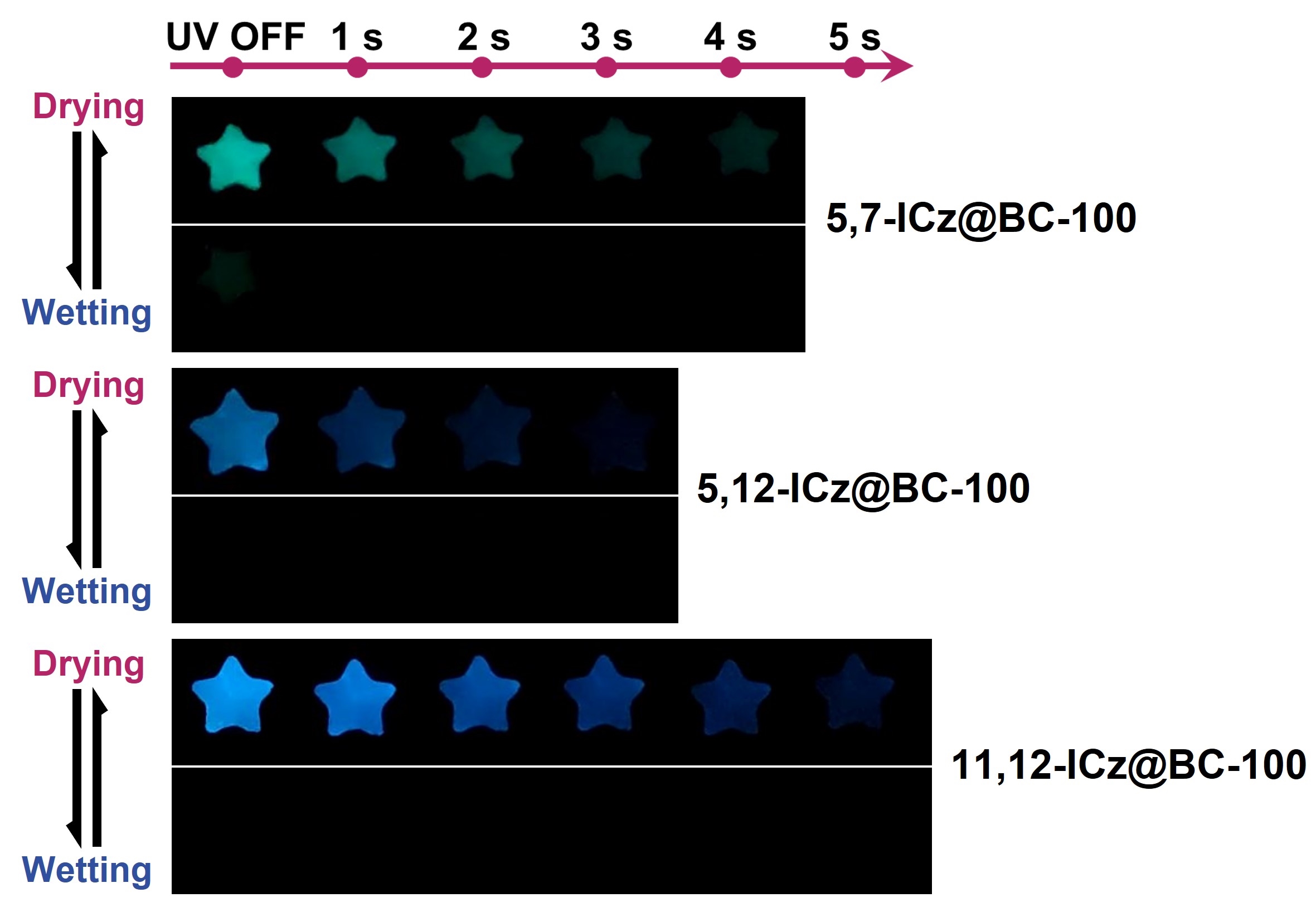


**Figure S15.** Photographs of the reversible wetting/drying process for **5,7-ICz@BC-100**, **5,12-ICz@BC-100**, and **11,12-ICz@BC-100** were taken under 365 nm UV light after ceasing the irradiation.


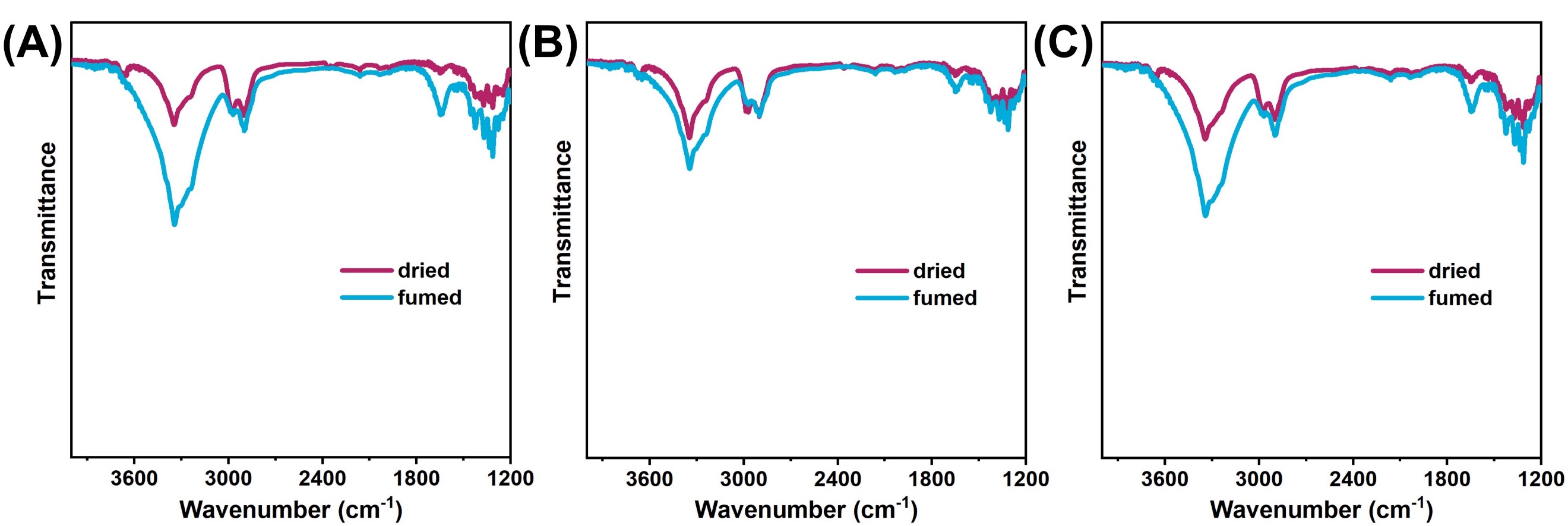


**Figure S16.** FT-IR spectra of (A) **5,7-ICz@BC-100**, (B) **5,12-ICz@BC-100**, and (C) **11,12-ICz@BC-100** under the dry/wet stimuli.


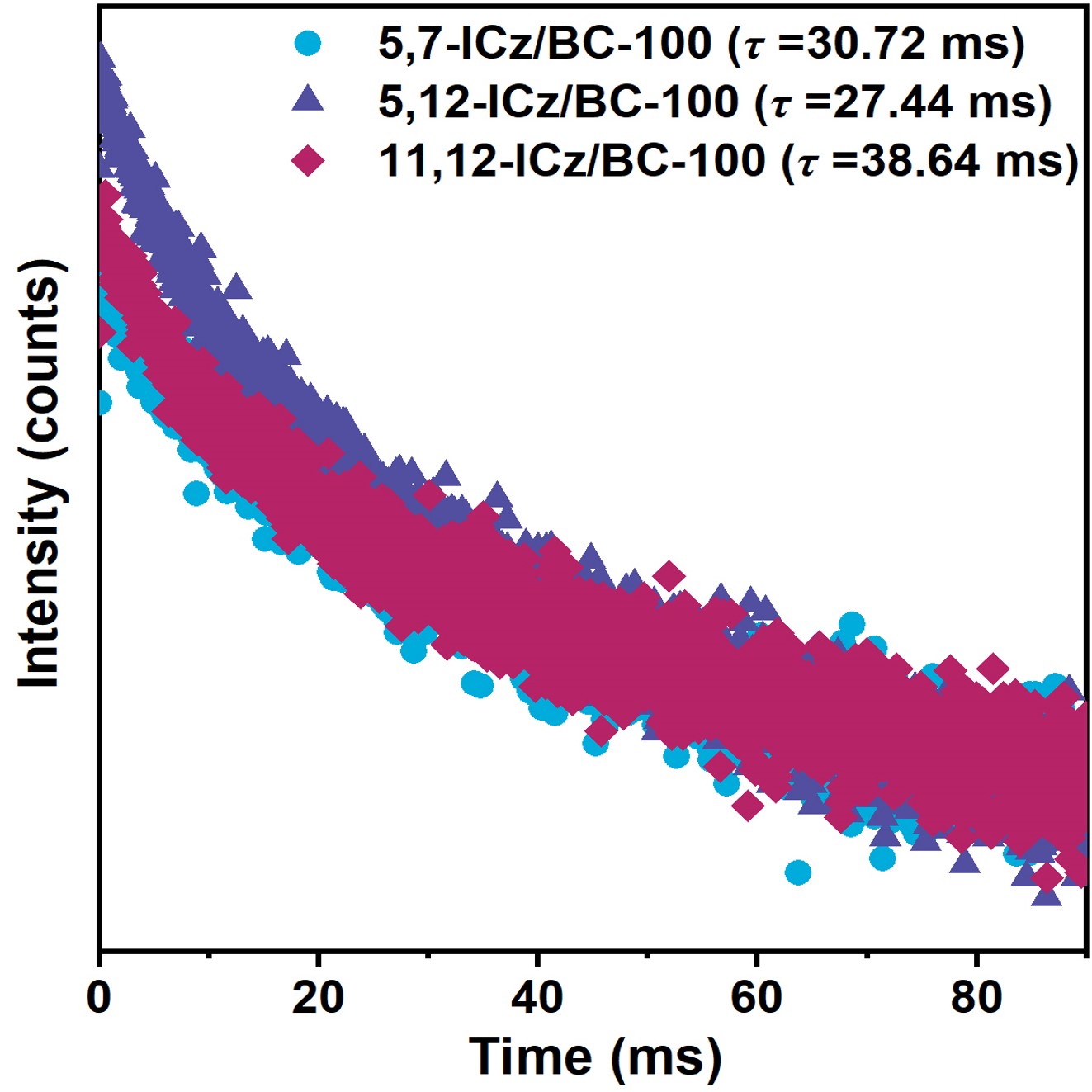


**Figure S17.** Time-resolved phosphorescent decay curves of **5,7-ICz/BC-100**, **5,12-ICz/BC-100**, and **5,12-ICz/BC-100**.


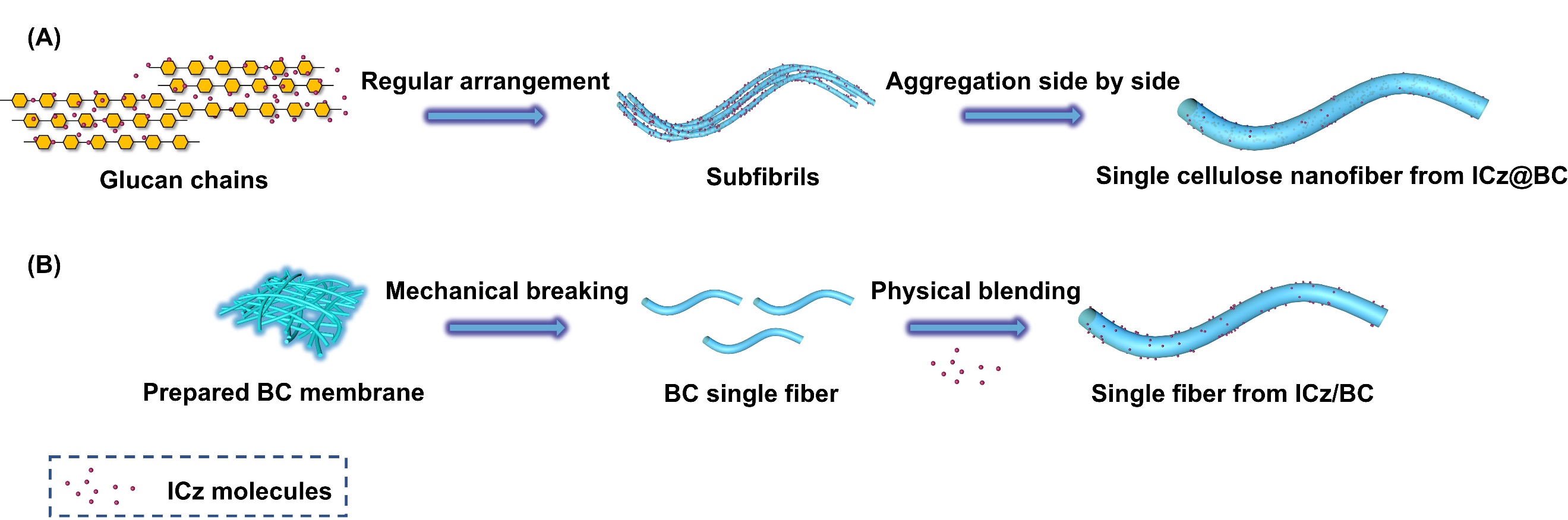


**Figure S18.** Proposed scheme illustration of (A) ICz@BC and (B) ICz/BC.


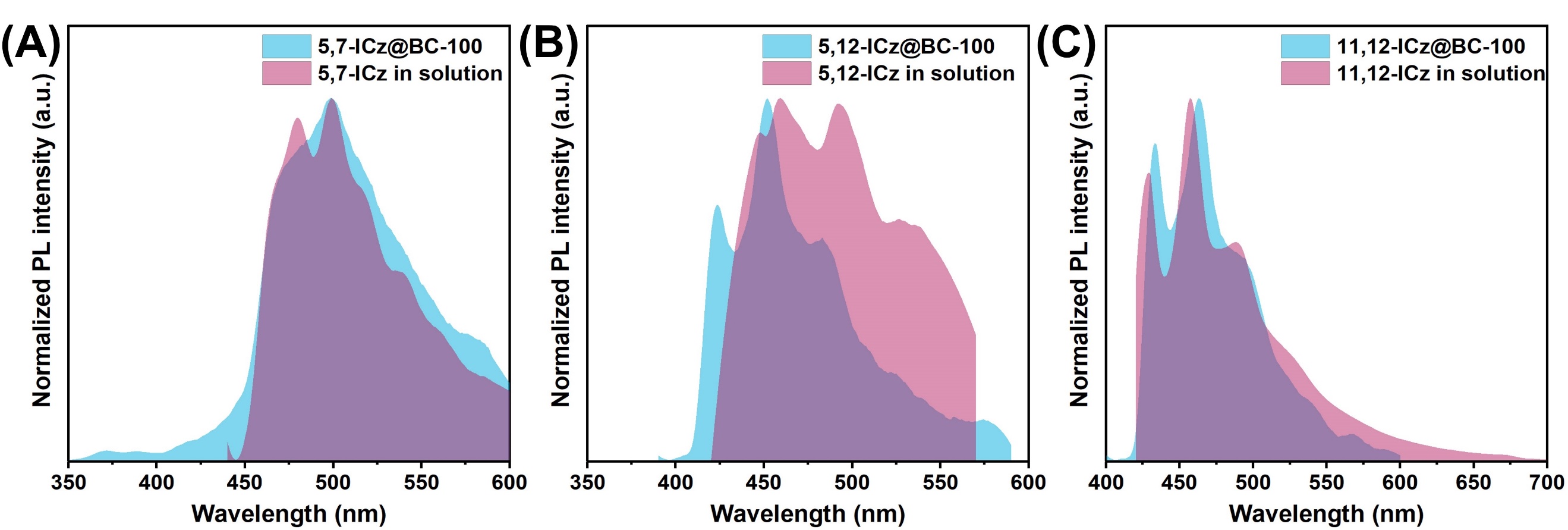


**Figure S19.** Delayed PL spectra of **5,7-ICz@BC-100**, **5,12-ICz@BC-100**, and **11,12-ICz@BC-100** and isomers in solution (10^-5^ M in THF at 78 K), respectively.


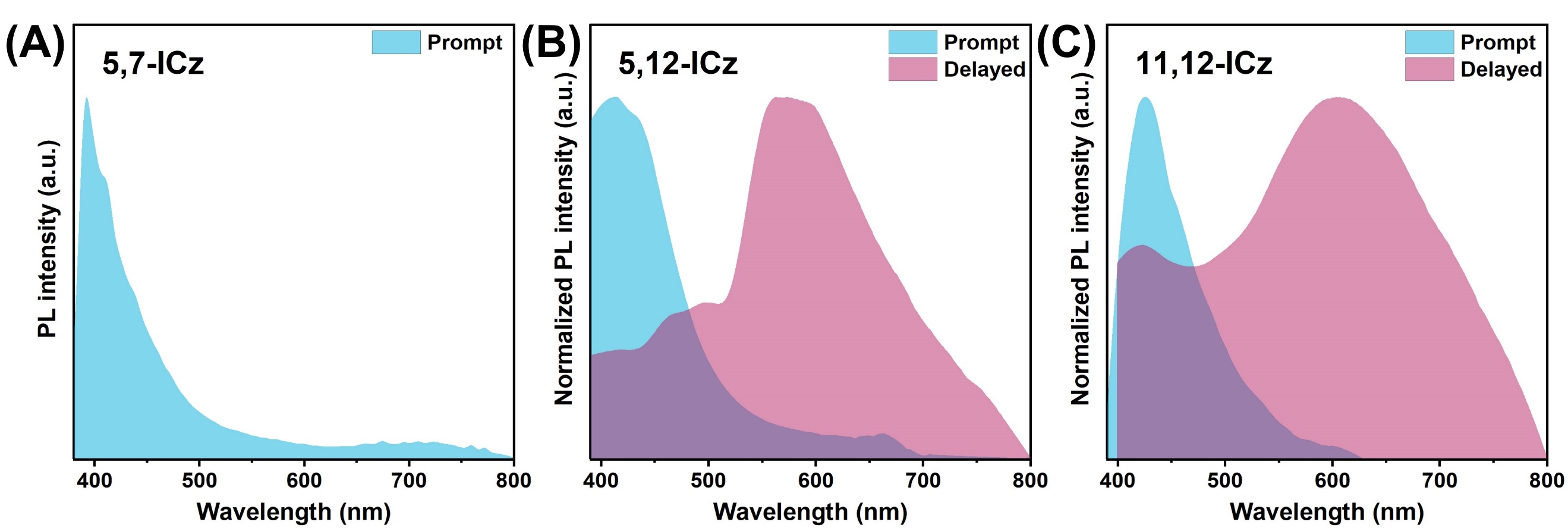


**Figure S20.** Prompt and delayed PL spectra of three ICz crystals.


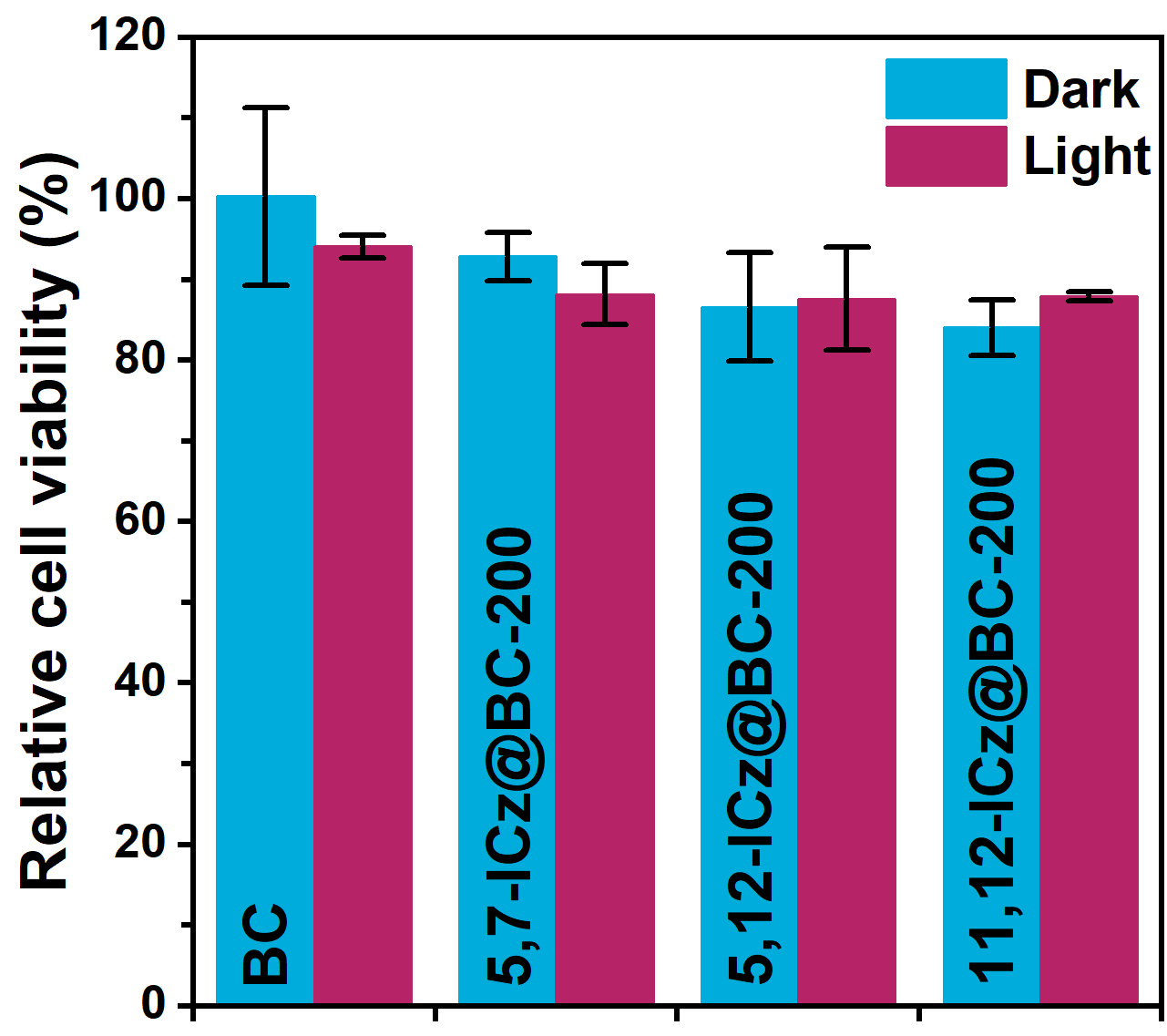


**Figure S21.** Relative cell viability of LO2 cells after 24 h of coincubation with **BC**, **5,7-ICz@BC-200**, **5,12-ICz@BC-200**, and **11,12-ICz@BC-200,** (n = 3; means ± SD).


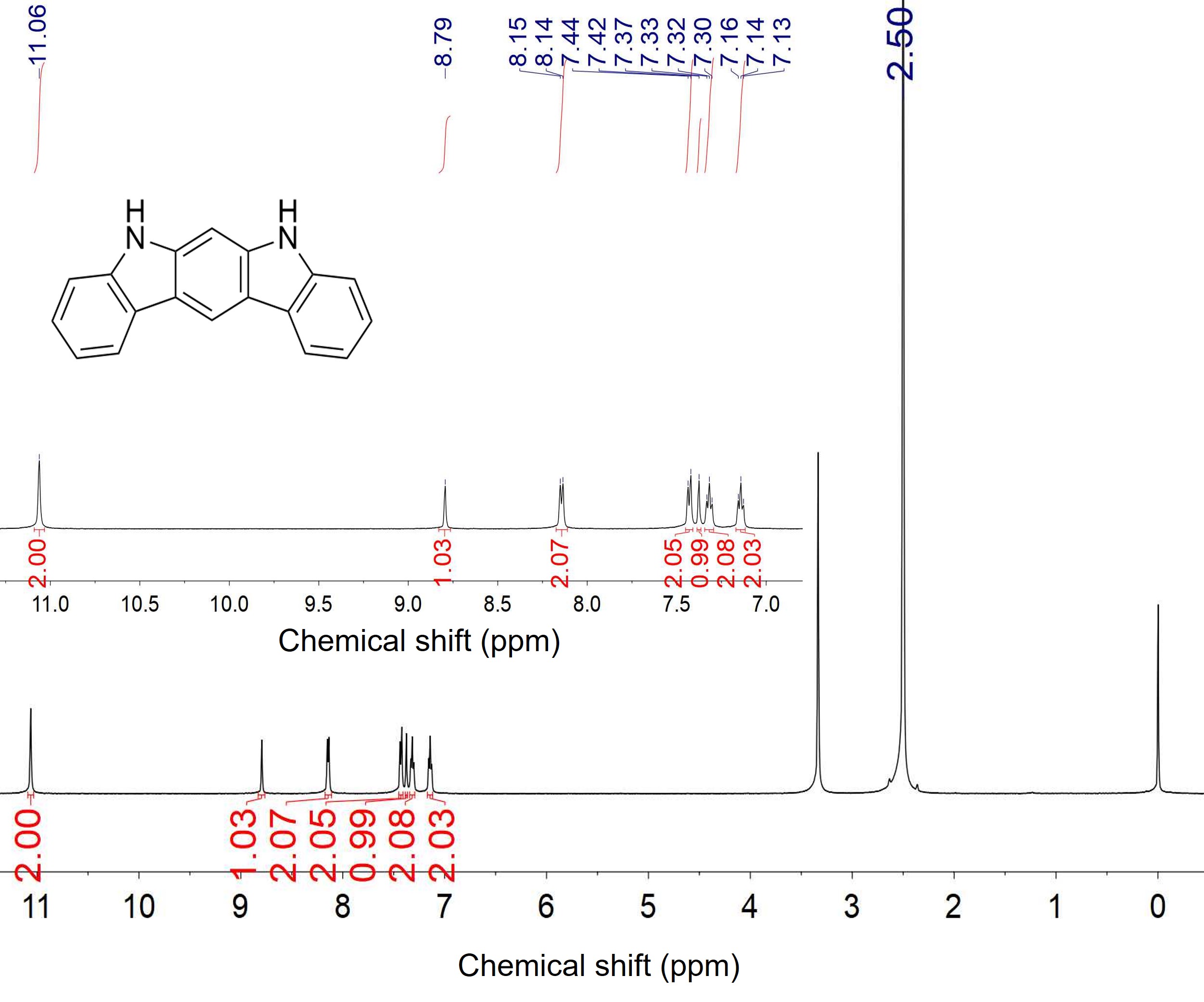


**Figure S22.** ^1^H NMR spectrum of **5,7-ICz** in DMSO-*d_6_*.


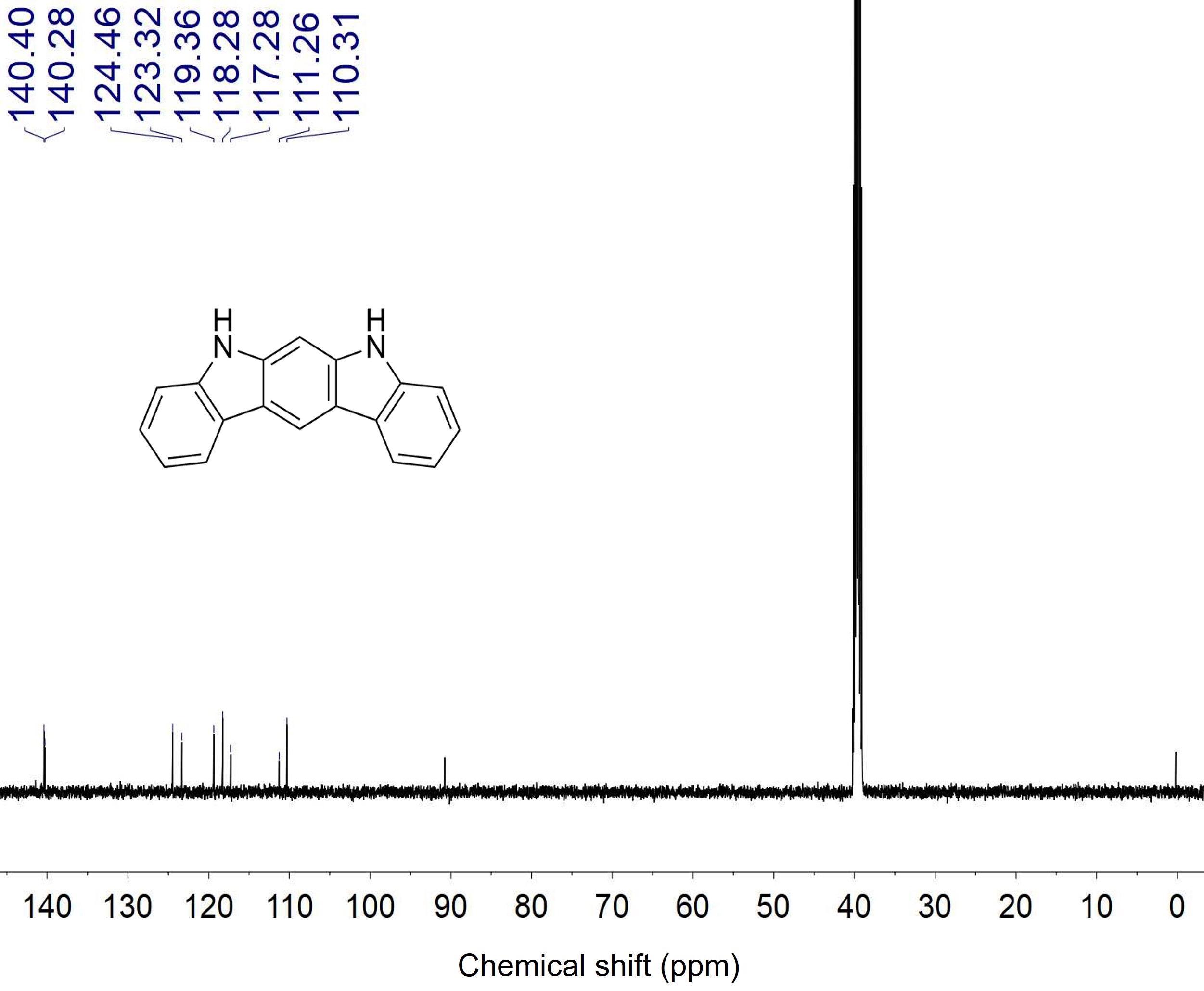


**Figure S23.** ^1^C NMR spectrum of **5,7-ICz** in DMSO-*d_6_*.


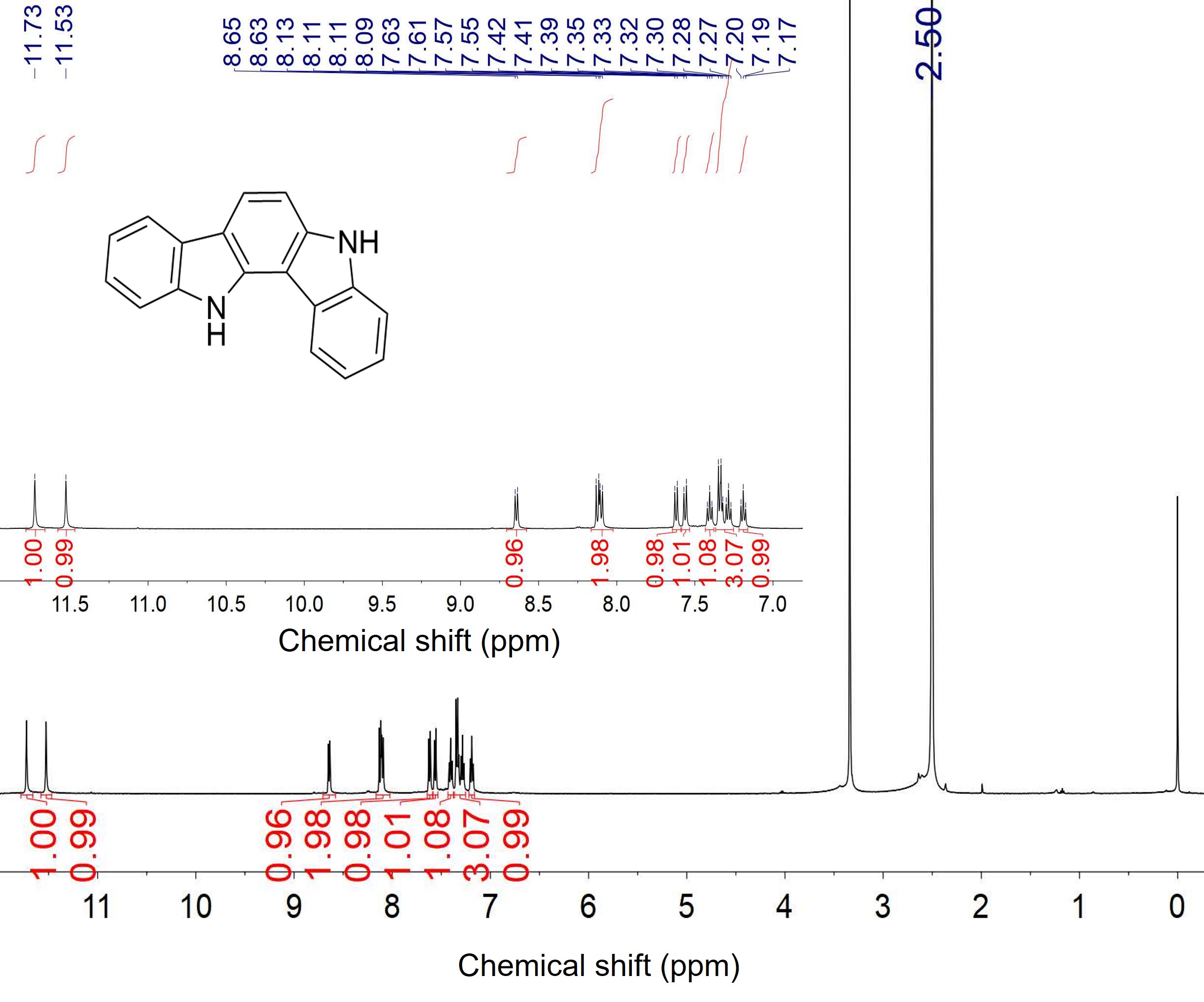


**Figure S24.** ^1^H NMR spectrum of **5,12-ICz** in DMSO-*d_6_*.


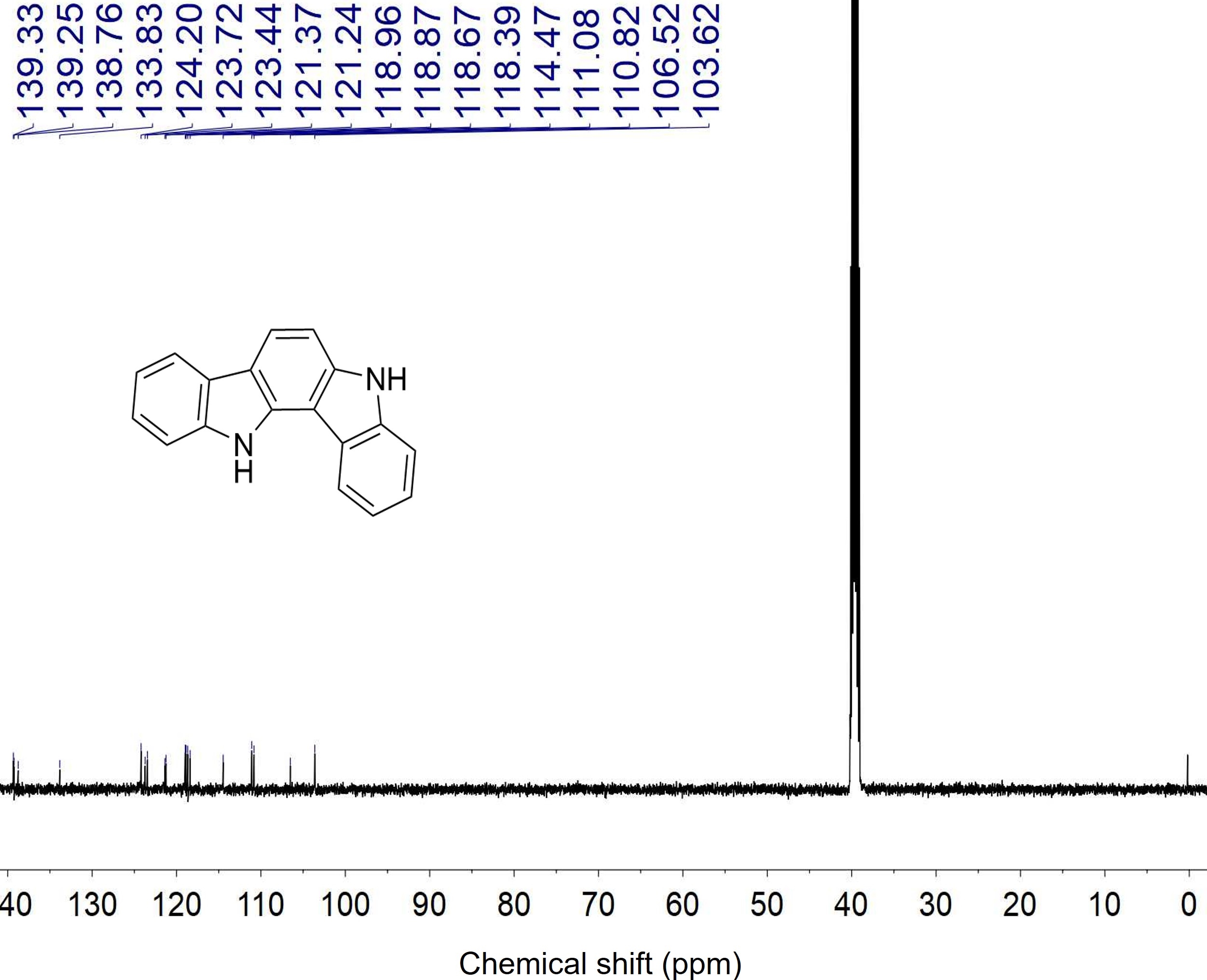


**Figure S25.** ^1^C NMR spectrum of **5,12-ICz** in DMSO-*d_6_*.


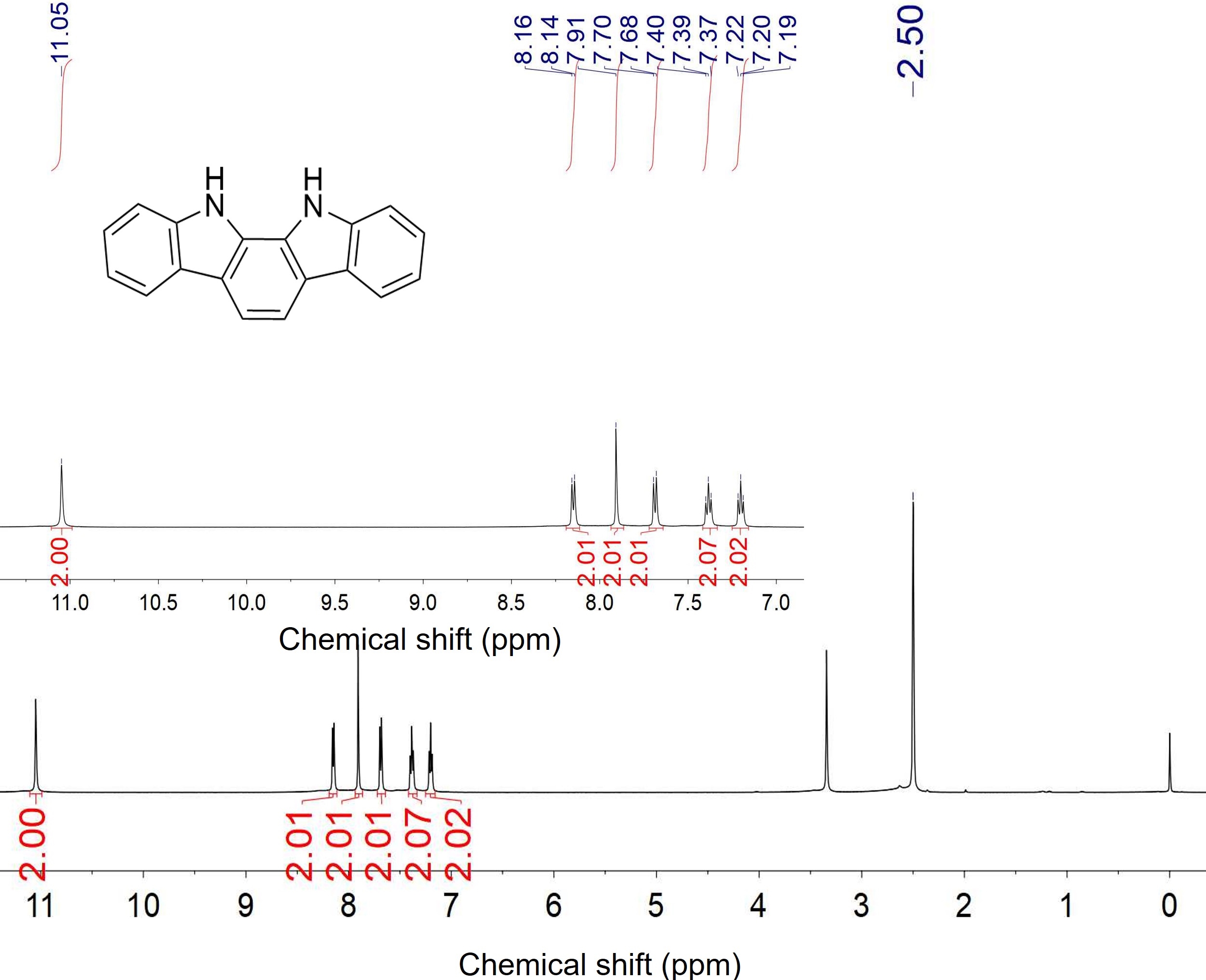


**Figure S26.** ^1^H NMR spectrum of **11,12-ICz** in DMSO-*d_6_*.


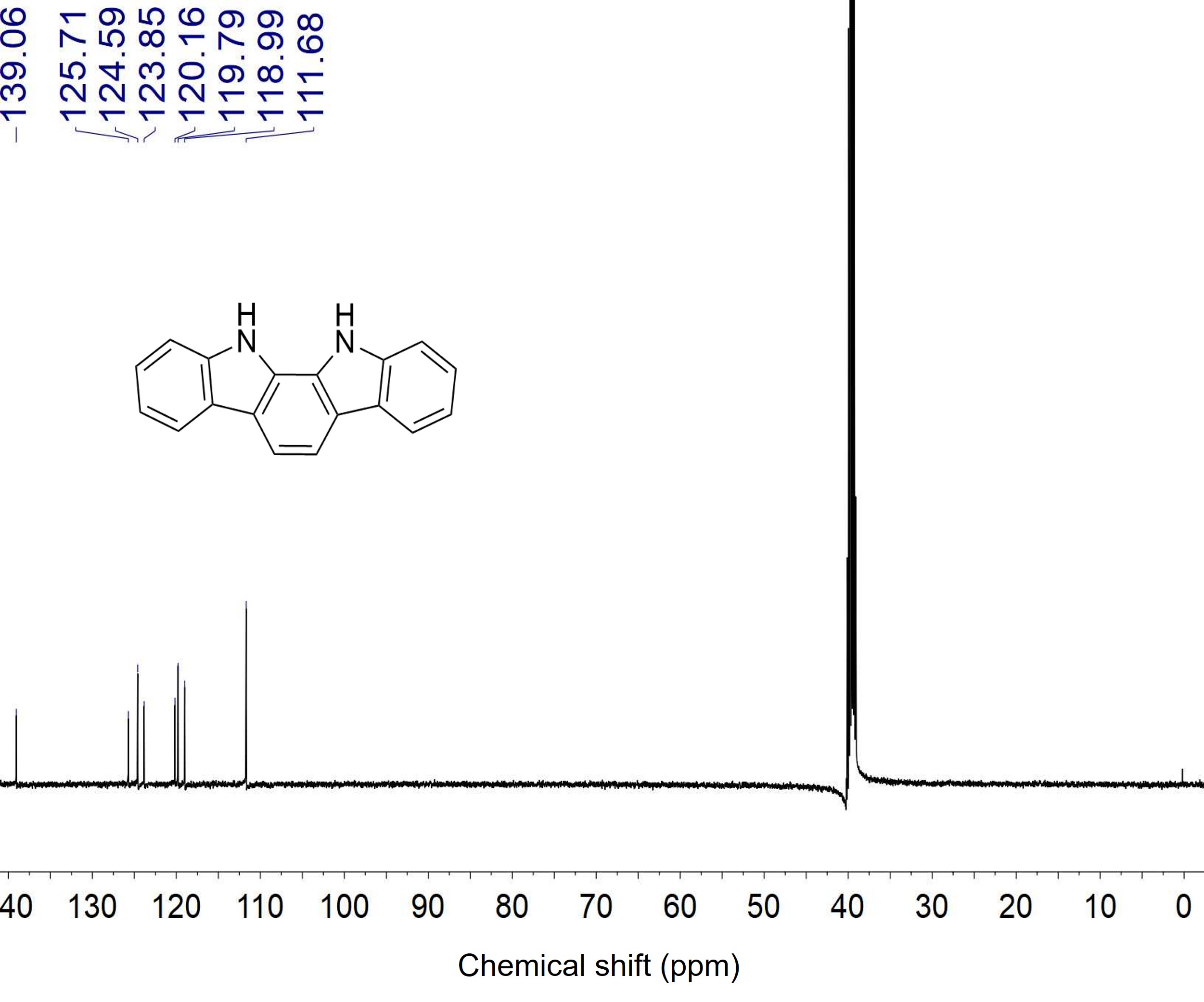


**Figure S27.** ^1^C NMR spectrum of **11,12-ICz** in DMSO-*d_6_*.

**Table S1.** Thicknesses of as prepared **BC** and **ICz@BC** samples (μm).

| **Samples** | **BC** | **5,7-ICz@BC-10** | **5,7-ICz@BC-50** | **5,7-ICz@BC-100** | **5,7-ICz@BC-200** |
| --- | --- | --- | --- | --- | --- |
| **Average diameter** | **44.67** | **62.33** | **51.33** | **65.67** | **45.33** |
| **Standard deviation** | **2.05** | **1.70** | **2.49** | **4.03** | **2.62** |

| **Samples** | **5,12-ICz@BC-10** | **5,12-ICz@BC-50** | **5,12-ICz@BC-100** | **5,12-ICz@BC-200** |
| --- | --- | --- | --- | --- |
| **Average diameter** | **53.67** | **56** | **56.33** | **56.67** |
| **Standard deviation** | **3.68** | **1.63** | **3.40** | **2.49** |

| **Samples** | **11,12-ICz@BC-10** | **11,12-ICz@BC-50** | **11,12-ICz@BC-100** | **11,12-ICz@BC-200** |
| --- | --- | --- | --- | --- |
| **Average diameter** | **52.33** | **46.33** | **49.67** | **56.67** |
| **Standard deviation** | **6.24** | **1.89** | **1.89** | **3.09** |

**Table S2.** RTP properties of the cellulose-based RTP materials from literature.

| Cellulose source | *λ*_em_ [nm] (prompt) | *λ*_em_ [nm] (delayed) | *τ*_P_ [ms] | Refs. |
| --- | --- | --- | --- | --- |
| Cationic cellulose derivative | - | 520 | 158 | [3] |
| CNC immobilized with cellulose-based phosphors | 461 to 493 | 499 to 626 | 654 | [4] |
| Anionic cellulose derivatives containing phenylcarboxylate groups | 435 | 512 | 433 | [5] |
| CDs@Cellulose | 460 | 560 | 167.31 | [6] |
| Hybrid chiral photonic  films designed by cellulose nanocrystals, poly(vinyl alcohol), and carbon dots | 450 to 470 | 480 to 500 | 103 | [7] |
| Cellulose grafted with aromatic amines | 425 | 530 | 571.1 | [8] |
| CNQDs-CMC film | 440 | 520 | 43 | [9] |
| FCDs-CMCNa | 550 | 550 | 270.8 | [10] |
| Lig⊂CNC | 365 | 520 | 103 | [11] |
| W-film | 320 | 500 | 241.9 | [12] |
| **5,7-ICz@BC-100** | **387** | **499** | **793.51** | **This work** |
| **5,12-ICz@BC-100** | **378** | **452** | **760.80** |  |
| **11,12-ICz@BC-100** | **385** | **464** | **858.05** |  |

**Table S3.** Calculated complexation energies and corresponding energy decomposing analysis (EDA) data between ICz molecules and cellulose (kcal/mol).

| ICz@BC systems | Electrostatic | Induction | Dispersion | Exchange | *E*_complex_ |
| --- | --- | --- | --- | --- | --- |
| **5,7-ICz@BC** | -24.81 | -8.01 | -23.47 | 30.88 | -25.41 |
| **5,12-ICz@BC** | -14.87 | -4.10 | -23.04 | 23.88 | -18.13 |
| **11,12-ICz@BC** | -24.17 | -7.51 | -22.95 | 27.67 | -26.96 |

**References**

[1] a) X. Nie, S. Wu, F. Huang, W. Li, H. Qiao, Q. Wang, Q. Wei, *Chem. Eng. J.* **2021**, *416*, 129072; b) X. Nie, P. Lv, S. L. Stanley, D. Wang, S. Wu, Q. Wei, *J. Appl. Polym. Sci.* **2019**, *136*, 48000.

[2] a) S. Emamian, T. Lu, H. Kruse, H. Emamian, *J. Comput. Chem.* **2019**, *40*, 2868-2881; b) D. Wang, H. Wu, J. Gong, Y. Xiong, Q. Wu, Z. Zhao, L. Wang, D. Wang, B. Z. Tang, *Mater. Horiz.* **2022**, *9*, 1081-1088.

[3] X. Zhang, Y. Cheng, J. You, J. Zhang, C. Yin, J. Zhang, *Nat. Commun.* **2022**, *13*, 1117.

[4] X. Zhang, C. Yin, J. You, R. Li, J. Zhang, Y. Cheng, Y. Wang, J. Zhang, *Research* **2023**, *6*, 0029.

[5] X. Zhang, J. You, J. Zhang, C. Yin, Y. Wang, R. Li, J. Zhang, *CCS Chem.* **2023**, *5*, 2140-2151.

[6] P. Wang, D. Zheng, S. Liu, M. Luo, J. Li, S. Shen, S. Li, L. Zhu, Z. Chen, *Carbon* **2021**, *171*, 946-952.

[7] M. Xu, X. Wu, Y. Yang, C. Ma, W. Li, H. Yu, Z. Chen, J. Li, K. Zhang, S. Liu, *ACS Nano* **2020**, *14*, 11130-11139.

[8] M. Zeng, T. Li, Y. Liu, X. Lin, X. Zu, Y. Mu, L. Chen, Y. Huo, Y. Qin, *Chem. Eng. J.* **2022**, *446*, 136935.

[9] Y. Kong, T. Lei, Y. He, G. Song, *Food Chem.* **2022**, *390*, 133135.

[10] M. Xu, C. Dong, J. Xu, S. ur Rehman, Q. Wang, V. Y. Osipov, K. Jiang, J. Wang, H. Bi, *Carbon* **2022**, *189*, 459-466.

[11] M. Cao, Y. Ren, Y. Wu, J. Shen, S. Li, Z. Yu, S. Liu, J. Li, O. J. Rojas, Z. Chen. *Nat. Commun.* **2024**, *15*, 2375.

[12] R. Liu, H. Guo, M. Cao, B. Dang, Y. Zhai, S. Liu, S. Li, J. Li, T. D. James, Z, Chen. *Adv. Funct. Mater.* **2023**, *34*, 2312254.
